# A study of size threshold for cooling effect in urban parks and their cooling accessibility and equity

**DOI:** 10.1038/s41598-024-67277-2

**Published:** 2024-07-13

**Authors:** Jun Zhang, Huina Zhang, Ruoming Qi

**Affiliations:** College of Landscape Architecture, Northeast Forestry University, Harbin, 150040 China

**Keywords:** Urban parks, Cooling effect, Threshold value of efficiency, Accessibility, Equity, Dominant factors, Environmental sciences, Environmental social sciences

## Abstract

Rapid urbanization has led to increasingly prominent urban heat island phenomena and social inequality. It is urgent to quantify the threshold area of urban parks from multiple perspectives to maximize the cooling effect and improve the equity of park cooling services. Using 33 urban parks in Harbin City as research objects, four indices, i.e., park cooling intensity (PCI), park cooling distance (PCD), park cooling area (PCA), and park cooling efficiency (PCE), were used to explore the park cooling effect and the threshold value of efficiency (TVoE) of the size. The OD (origin–destination) matrix model was constructed to assess the spatial accessibility from the community to the cooling range. The Gini coefficient was used to assess the equity of cooling range accessibility. The relative contribution of each influencing factor to the cooling indicator was quantified through regression modeling. The results showed that the average PCI was 3.27 ℃, the average PCD was 277 m, the average PCA was 115.35 ha, and the average PCE was 5.74. Gray space area was the dominant factor for PCI, PCD, and PCA (relative contributions of 100%, 31%, and 19%, respectively). Park area was the dominant factor for PCE (relative contribution of 28%). The TVoE of park sizes based on PCA and PCE were calculated as 82.37 ha and 2.56 ha, respectively. 39.2% and 94.01% of communities can reach cooling ranges within 15 min in walk mode and transit mode, respectively. Approximately 18% of neighborhood residents are experiencing severe inequities in cooling range accessibility. This study can guide park design that maximizes cooling effects, as well as inform city planners on more equitable allocation of urban park resources.

## Introduction

Rapid urbanization not only increases the number of people within cities but also changes the urban surface cover, such as by increasing impervious surfaces and decreasing natural vegetation^1^. These changes have exacerbated the urban heat island (UHI) phenomenon, causing urban land surface temperatures (LST) to be higher than suburban LST^2,3^. Temperatures in urban areas are projected to increase from 2.5 to 4.5 °C by the end of the twenty-first century from the UHI effect^4^. A growing body of evidence suggests that continued urban warming seriously threatens the sustainability of cities and affects the thermal comfort of urban residents. This may increase mortality rates, especially during extreme heat events^5–7^. The UHI effect has become a huge challenge for cities around the globe. Therefore, mitigating the UHI effect has become a global concern^8^.

Numerous studies have shown that urban parks, as the most common blue-green landscape combination space in cities, can reduce LST to a certain range^9,10^. Researchers assessed LST in 86 urban parks in Barcelona and found that 84 had a cooling effect^11^. Many scholars to date have proposed various indices with a temperature reduction effect. Yu et al. proposed and defined the extent, intensity, and efficiency of urban cold islands^12^. Other studies have proposed cooling indices such as cooling intensity, cooling gradient, cooling distance, and cooling efficiency^9^. Researchers evaluated the cooling effect of 384 parks in five U.S. cities and found that the cooling effect varied significantly between cities^13^. The maximum cooling intensity in Addis Ababa city park was 6.72 °C and the maximum cooling distance was 240 m^14^. The average cooling intensity of urban parks in Beijing City was 1.71 ℃, and the average cooling gradient was 0.74 ℃/hm^15^. The average cooling intensity of urban parks in Wuhan City was 3.5 ℃, the average cooling area was 131.6 ha, the average cooling gradient was 17.9 ℃/km, and the average cooling efficiency was 4.5^16^. Several studies have shown that park size plays an important role in the cooling effect^9^. The cooling area is positively correlated with park size, and parks with larger cooling areas are mostly large parks^17^. The cooling efficiency is negatively related to park size, the smaller the park size, the higher the cooling efficiency. Other studies have shown that there is no significant correlation between park size and cooling intensity and that some small parks can achieve the same cooling intensity effect as some large parks^16^. Park size was found to be the dominant factor affecting cooling intensity in Geng et al.'s study, explaining more than 50% of the variation in cooling intensity^18^. Other scholars have found that the area of urban parks has a nonlinear relationship with the cooling effect^19–21^. If the park area exceeds a threshold value, the cooling effect will be significantly reduced. To obtain the optimal cooling efficiency, Yu et al. proposed the concept of a threshold value of efficiency (TVoE) from the perspective of the "law of diminishing marginal utility"^12^. Shi et al. suggested the range of urban parks' construction area from 13.3 to 19.4 ha according to the different urbanization levels^17^. Geng et al. proposed that the TVoE of urban parks was 0.81 ha, 0.71 ha, 0.70 ha, and 0.66 ha under four different climatic backgrounds in East China^18^. The TVoE of parks in Chengdu, Fuzhou, and Taipei were 30 ha, 1.08 ha, and 3 ha, respectively^22–24^. The TVoE of urban parks varies depending on the cities and climatic contexts. Thus, more research is needed. In addition, the cooling effect is simultaneously affected by the urban park's landscape characteristics and the surrounding landscape configuration^25^. A study in Perth, Australia, showed spatial differences in the cooling effect of different vegetation types. Shrubs and trees had a greater cooling effect than grass^26^. A study in Nagoya, Japan, concluded that the cooling intensity of a park is mainly determined by the area of trees in the park and the shape of the park, and that grass has a negative effect on the formation of cooling intensity^27^. Du et al. concluded that the higher the normalized difference vegetation index on the exterior of the park, the lower the cooling efficiency of the park^25^. Chen et al. found that normalized difference vegetation index in parks positively correlated with cooling area and cooling intensity and is not correlated with cooling gradient and cooling efficiency^16^. Yao et al. found that the normalized difference vegetation index had a significant correlation with the cooling area but not with any other cooling indices^24^. Xie et al. found a positive correlation between the area of water bodies and the cooling intensity in Wuhan parks, suggesting that for every 10% increase in the proportion of water body area, the cooling intensity increases by about 0.5 ℃^28^. A study in Beijing showed that the proportion of water body area within the park was positively correlated with cooling intensity. The proportion of water bodies in the buffer zone outside the park was not correlated with cooling intensity^15^. Other scholars found that the proportion of water bodies in parks was positively correlated with the intensity of cooling in the warm temperate semi-humid monsoon climate, and the relative contribution to the intensity of cooling was 13.24%. The proportion of water bodies is not correlated with the cooling intensity under the background climatic conditions of the Northern subtropical semi-humid monsoon climate, Northern subtropical humid monsoon climate, and Central subtropical humid monsoon climate^18^. In addition, a study by Peng et al. in Shenzhen showed that the area of water bodies did not significantly affect the cooling index of parks^9^. It can be observed that the influence of landscape features inside and outside the park on the cooling effect needs to be further explored.

In addition, most researchers have paid more attention to the cooling effect of parks and little attention to the spatial accessibility and equity of the cooling range of parks^17,29^. China is the largest developing country and has experienced rapid urbanization in recent decades. Rapid economic and social development has led to a surge in urban population, gradually exacerbating the conflict between social resource supply and population demand. With the cooling demand of UHI effect and unequal resource supply under rapid urbanization, it is crucial to assess the spatial accessibility and equity of the cooling range of urban parks, which in turn guides more effective cooling supply and environmental justice.

In summary, related studies have examined the cooling effect of urban parks from different perspectives, such as the cooling index, TVoE, and influencing factors^30–32^. We found significant differences in the conclusions reached in different urban climate contexts. Most of the current evaluation studies have been conducted in very hot climates in China. There are very limited studies conducted in the cold regions of China. Yang et al.'s study on urban green spaces in Changchun City, a severe cold region, showed that the cooling effect of urban green spaces was mainly influenced by the normalized difference vegetation index and the area of urban green spaces^33^. In another study by Yang et al., it was found that urban parks can form park cold islands^34^. The study showed that park cold islands were positively correlated with park size, park perimeter, and percentage of water area. The park cold island effect did not increase dramatically when the park threshold area reached 80 ha. Huang et al. investigated the cooling effect of urban green space in Harbin City in a severe cold region and quantified the cooling intensity and cooling distance of urban green space^35^. They showed that increasing the green area up to 37 ha can improve its cooling effect with minimum economic cost. The above study verified the cooling capacity of urban parks or green spaces in severe cold regions in terms of mitigating the urban heat island phenomenon. Despite the contribution of previous studies, there are still shortcomings in the literature. Firstly, the relevant literature mainly focuses on the correlation analysis between the landscape characteristics of parks or green spaces and the cooling effect. There is a lack of exploration of the dominant characterizing factors and their relative contribution to the cooling effect. Secondly, these studies only explored the influence of landscape features (area, perimeter, etc.) inside the green space or park on the cooling effect, and did not consider the influence of landscape features outside the park or green space (proportion of green landscape in the buffer zone, etc.) on the cooling effect. Then, due to the scarcity of urban land resources, the threshold area of parks under the maximization of cooling effect needs to be urgently addressed. Most of the studies calculated the threshold area based on one indicator is not comprehensive. TVoE should be quantitatively explored from multiple perspectives. In addition, the spatial accessibility and equity of the cooling range of parks are seldom evaluated when studying the cooling effect of parks.

To solve the above problems, this study takes Harbin City, Heilongjiang Province, a severe cold region in China, as an example. Firstly, to fill the research gap of the cooling effect of urban parks in Harbin City during the summer in severe cold regions. Because the previous relevant studies did not consider the influence of external landscape features on the cooling effect. In this study, the influence of internal landscape features and external landscape features on the cooling effect of parks was jointly discussed. Secondly, we identified the dominant factors affecting the cooling effect. The relative contribution of each factor to the overall explanation of changes in cooling indicators was quantified. Then, previous studies were based on a single indicator to calculate the TVoE for the size of a park or green space. In this study, we quantified the TVoE of urban park size from multiple perspectives of cooling area and cooling efficiency, respectively. In addition, previous studies have paid little attention to the accessibility and equity of park cooling ranges. In this study, the accessibility of cooling range was calculated for walking and public transportation modes. It also assesses the equity of residents' access to cooling services using neighborhoods as spatial units. We organized 33 urban parks and quantified their cooling effects in summer. The objectives were to (1) quantify the cooling effect of urban parks; (2) identify the dominant internal and external landscape features of the park and their relative contribution to the cooling effect; and (3) quantify the TVoE of urban park size based on the cooling area index and cooling efficiency index, respectively; (4) the accessibility and equity of the park cooling area was assessed. In cities with scarce land resources, the study not only explored the optimal park area for maximizing the cooling effect, but also provided a reference for improving the equity of social resources and optimizing the rational allocation of park resources.

## Materials and methods

### Study area and data

**Figure 1:**
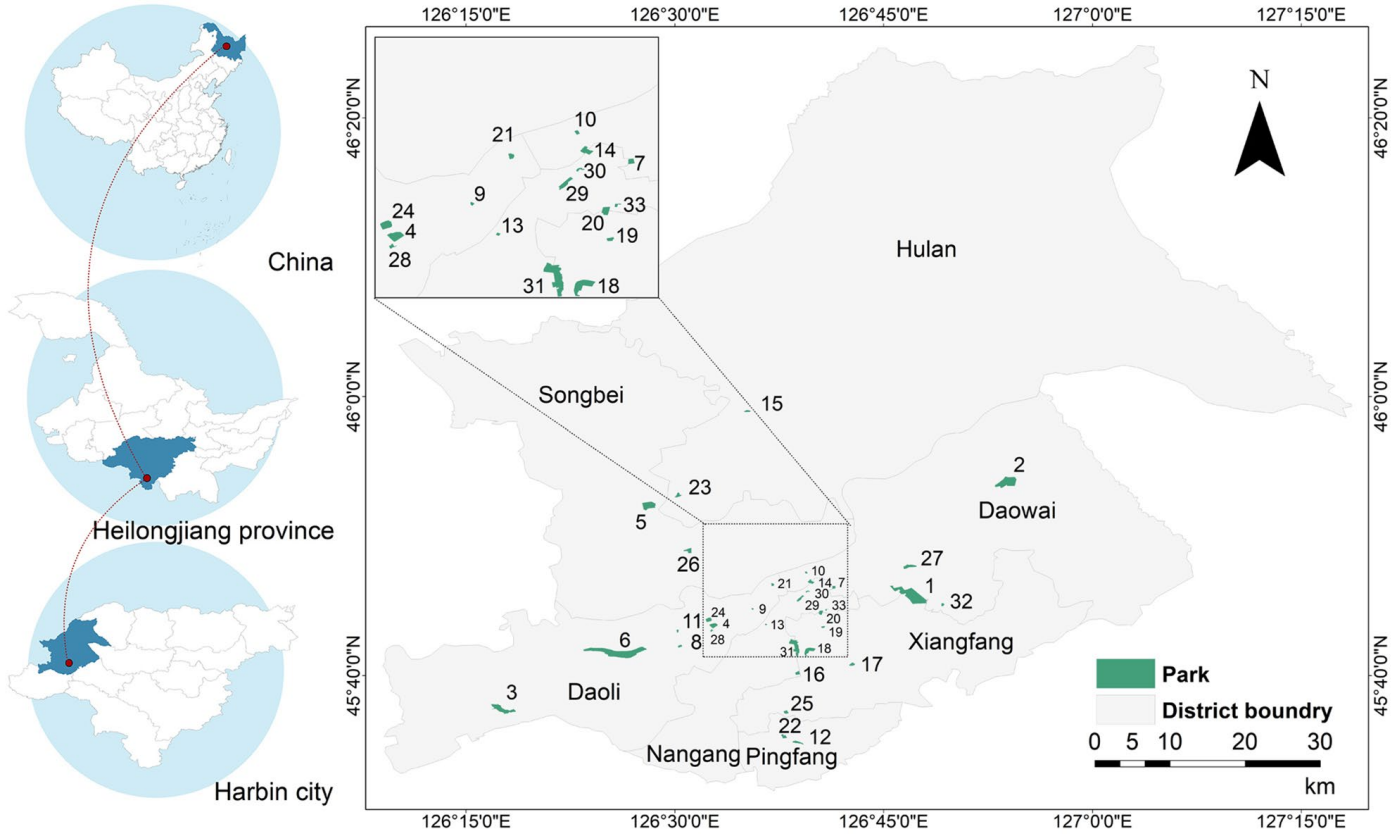
Research location and distribution map of urban parks in Harbin. Created using ArcGIS software and Adobe Illustrator. The base map was from the standard map with review number GS (2020) 4619 downloaded from the website of Standard Map Service of the Ministry of Natural Resources, with no modification of the base map (http://bzdt.ch.mnr.gov.cn/).

Harbin is the capital city of Heilongjiang Province and an important center city in northeast China. The geographic location is 125°42′ E–130°10′ E, 44°04′ N–46°40′ N (Fig. 1). It has a mid-temperate continental monsoon climate, with long winters and short summers, and four distinct seasons. The average temperature in winter is about −19 ℃, and the average temperature in summer is about 23℃^35^. The average precipitation throughout the year is 569.1 mm, mainly concentrated in June–September, and the number of frost-free days is 168 days. According to the 2021 Harbin Statistical Annual Signature data (Harbin Municipal Government Website https://www.harbin.gov.cn), the resident population and urbanization rate of Harbin in 2020 were 10.01 million and 70.6%, respectively. We chose to study the highly urbanized main urban area of Harbin, including the seven administrative districts of Daoli, Daowai, Nangang, Songbei, Hulan, Xiangfang, and Pingfang, which have a high intensity of development. Rapid urbanization has led to severe UHI effects, and the study area has faced high population and severe thermal environmental problems in recent years.

Based on previous studies, the following principles were considered for the selection of park samples: (1) considering the limitations of Landsat 8 imagery data resolution, parks with an area greater than 0.09ha were selected^36^; (2) selection of parks that are more than 600 m away from other large green spaces or water bodies to avoid other large blue-green spaces interacting with the study samples^9,36^; (3) the land cover type in the parks was dominated by vegetation or water bodies; and (4) parks with significant differences in area and shape were selected. Based on these principles and following the "Basic Information on Wetland Parks in Harbin City" and "Public Information Content of Urban Parks and Scenic Spots in Harbin City" officially released by the Harbin City Government in 2018 (Harbin City Government Website http://www.harbin.gov.cn), we selected 33 urban parks in the study area for this study (Fig. 1). Among them, 13 parks contain water bodies. The minimum park area is 2.89 ha, the maximum park area is 381.17 ha, and the average area is 45.67 ha. This study is based on the Baidu map and uses ArcGIS for manual visual interpretation to identify and digitize the boundaries of urban parks.

Previous studies have demonstrated that LST inverted from remotely sensed imagery can effectively quantify the cooling effect of urban parks^15,24,25^. Therefore, we searched for remotely sensed imagery from 2017 to 2022, with imaging from late June to early September. All other images were cloudy and the inversion could not be completed. Two Landsat 8 OLI/TIRS images (path 118, row 28) acquired from the U.S. Geological Survey (USGS, https://earthexplorer.usgs.gov) were finally used as raw data in this study. The acquisition dates are July 7, 2017, and September 4, 2021, respectively. The remote sensing image acquisition dates were all sunny, with 0 rainfall and wind speeds less than 4 m/s. The mean air temperatures were 24 °C and 22 °C, respectively. The cloudiness of the study area was 0, which satisfied the LST inversion requirements. Land cover data and vegetation type data were obtained from the 2021 ESA World Cover product (https://viewer.esa-worldcover.org/worldcover/) at a resolution of 10 m. In the global land cover product, we identified trees, grasslands, scrub, and cropland as green spaces and waters and herbaceous wetlands as blue spaces. Buildings are classified as gray spaces, and the remaining land is considered other spaces^19^. Built-up volume data was obtained from the 2020 GHSL (Global Human Settlement Layer) product (https://human-settlement.emergency.copernicus.eu/) at a resolution of 90 m. City roadway network data was obtained from the Open Street Map (http://www.openstreetmap.org). Neighborhood data was obtained from the Anchorage website (https: //www.anjuke.com/).

### LST retrieval

There are more LST inversion algorithms at present. Compared with other algorithms, the radiative transfer equation algorithm calculates the acquired LST with higher accuracy, and the inversion results can meet the needs of studying urban parks^24,37^. Therefore, in this study, ENVI software was applied, and the radiative transfer equation algorithm was used to calculate LST.

The atmospheric influence on surface radiation is first estimated. Then, this atmospheric influence is subtracted from the total thermal radiation observed via satellite remote sensing to obtain the surface radiation intensity. Finally, this radiation intensity is converted to the corresponding LST with the following equation^37–39^.

1 $${L}_{\lambda }=[\begin{array}{c}\varepsilon {\text{B}}({T}_{s})+(1-\varepsilon ){L}_{atm,i}\downarrow \end{array}]\tau +{L}_{atm,i}\uparrow $$

2 $$B({T}_{s})=[\begin{array}{c}{L}_{\lambda }-{L}_{atm,i}\uparrow -\tau (1-\varepsilon ){L}_{atm,i}\downarrow \end{array}]/\tau \varepsilon $$

where ${\text{L}}_{\uplambda }$ is the thermal infrared radiation brightness received via the satellite sensor, ${\text{L}}_{\text{atm},\text{i}}\downarrow$ is the downward radiation luminance of the atmosphere, ${\text{L}}_{\text{atm},\text{i}}\uparrow$ is the upward radiation luminance of the atmosphere, $\text{B}({\text{T}}_{\text{s}})$ is the blackbody thermal radiant luminance, ${\text{T}}_{\text{s}}$ is the LST, $\upvarepsilon$ is the surface-specific emissivity, and $\uptau$ is the atmospheric transmission rate^38^. The atmospheric transmittance $\uptau$, atmospheric downward radiant brightness ${\text{L}}_{\text{atm},\text{i}}\downarrow$ , and atmospheric upward radiant brightness ${\text{L}}_{\text{atm},\text{i}}\uparrow$ in Eq. (2) are available through the official NASA website (http://atmcorr.gsfc.nasa.gov).

3 $${T}_{s}=\frac{{K}_{2}}{\mathit{ln}(\frac{{K}_{1}}{B({T}_{s})}+1)}$$

where ${\text{K}}_{1}({\text{Wm}}^{-2}{\text{sr}}^{-1}\upmu {\text{m}}^{-1})$ and ${\text{K}}_{2}(\text{K})$ are radiation constant. ${\text{K}}_{1}$ = 774.89, and ${\text{K}}_{2}$ = 1321.08^39^.

### Quantification of park cooling effect

**Figure 2:**
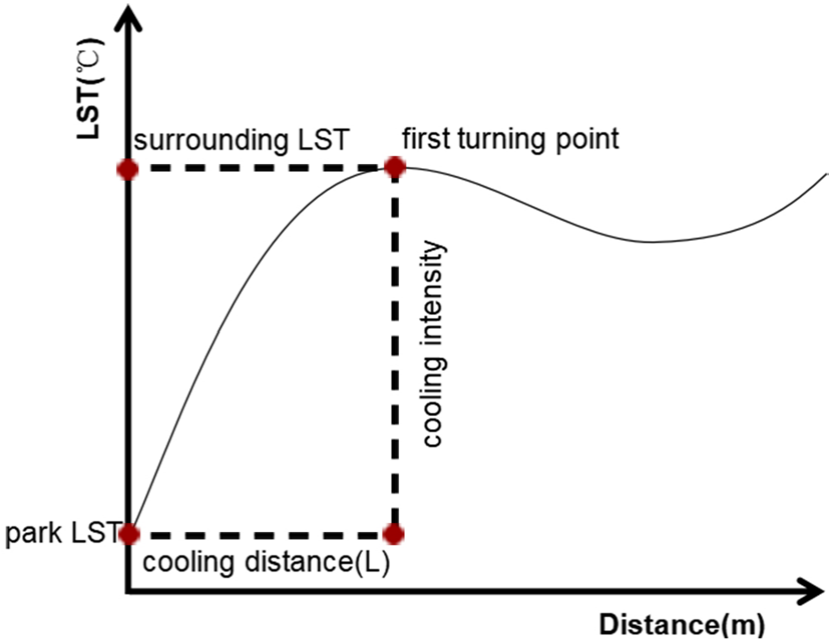
The sketch map of the park cooling curve.

Based on previous studies, considering the spatial resolution of Landsat 8 imagery (30 m) and the limited cooling distance of urban parks, a 600-m wide buffer zone was created from the boundary of each urban park and subdivided into twenty 30-m wide buffer zones to quantify the cooling effect of the urban parks^9,24^. Then, the ArcGIS zoning statistics tool was used to count the average temperature of each buffer zone^18^. To further determine the scope and extent of the influence of different parks on the surrounding temperature, a fitting analysis was conducted with the distance of the buffer zone as the independent variable $\text{r}$ and the average temperature within the buffer zone as the dependent variable $\text{T}$ ^40^. The average temperature within the buffer zone was then calculated using the ArcGIS partitioning statistics tool. Based on previous studies, the degree of fit of the cubic polynomial is high, and the fitting polynomial is shown in Eq. (4) ^41^. An image of the mean LST in the park buffer zone as a function of distance was plotted with buffer zone temperature as the vertical axis and buffer zone distance as the horizontal axis (Fig. 2).

4 $$T(r)=a{r}^{3}+b{r}^{2}+cr+d$$

As shown in Fig. 2, the greater the distance from the park boundary, the more the LST increases. However, after the distance increases to a certain point, the LST flattens out until the slope of the curve is 0. This point is called the first turning point of the curve^42^. If the cubic polynomial does not have a point with a derivative of 0, then the point with the smallest value of the derivative is used as that turning point. The cooling effect of the park will disappear if greater than this distance, and the distance corresponding to the turning point is the park cooling distance (PCD)^16^. Park cooling area (PCA) is the maximum influence range of the cooling effect of parks, which is determined by the PCD^43^. Park cooling efficiency (PCE) is the ratio of PCA and park area, characterizing the cooling area per unit area of urban parks and reflecting the economy of parks in generating cooling effects^43^. Park cooling intensity (PCI) is the difference between the LST at the first turning point (PCD) and the LST inside the park^16,44^. Finally, this study used PCI, PCD, PCA, and PCE as the evaluation indexes of the cooling effect in urban parks.

### Factors affecting park cooling effect

**Figure 3:**
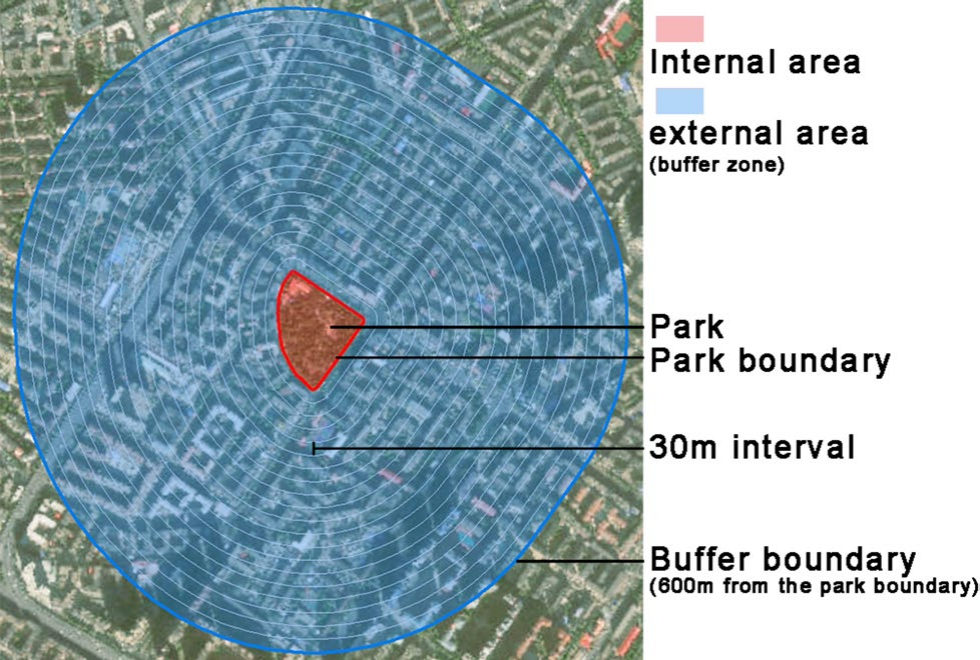
Schematic diagram of the park research area.

Factors affecting the cooling effect of the park can be categorized into internal and external factors^15^. Based on the more widely applied influencing factors in previous studies, we selected 13 indicators from three aspects: vegetation type, landscape composition and shape (Table 1). Internal factors include the area of trees (TR), the area of grassland (GR), the area of shrubland(SH), area, perimeter, landscape shape index (LSI), the area of blue space (Blue), the area of green space (Greenin), and normalized difference vegetation index (NDVIin). External factors included the built-up volume in the buffer zone (BV), the area of green space in the buffer zone (Greenout), the area of gray space in the buffer zone (Grey), and normalized difference vegetation index in the buffer zone (NDVIout ). A schematic of the park study area is shown in Fig. 3. The formula for the LSI was as follows.

5 $$LSI=\frac{P}{2\sqrt{\pi \times {S}_{park}}}$$

where P represents the perimeter of the park, and ${\text{S}}_{\text{park}}$ represents the size of the park. LSI characterizes the complexity of park shapes.

**Table 1:** Selection of influencing factors.

Factor classification	Factors	Description
Landscape characteristics of the park itself	Park area (area)	The total area of each park
	Park shape length (perimeter)	The perimeter of each park
	Landscape shape index (LSI)	The landscape shape index of each park
	The area of blue space (Blue)	The area of blue space in each park
	The area of green space (Greenin)	The area of green space in each park
	Normalized difference vegetation index (NDVIin)	The normalized difference vegetation index of each park
	The area of trees (TR)	The area of trees in each park
	The area of grassland (GR)	The area of grassland in each park
	The area of shrubland (SH)	The area of shrubland in each park
Landscape characteristics around the park	The area of green space in the buffer zone (Greenout)	The area of green space in the 600 m buffer of each park
	The area of grey space in the buffer zone (Grey)	The area of grey space in the 600 m buffer of each park
	Normalized difference vegetation index in the buffer zone (NDVIout)	The normalized difference vegetation index in the 600 m buffer of each park
	The built-up volume in the buffer zone (BV)	The built-up volume in the 600 m buffer of each park

### Identification of different cooling bundles

In this study, the SPSS hierarchical clustering method was applied to classify urban parks. Firstly, the four cooling indices are normalized. Then, the parks are divided into different cooling capacity bundles according to the numerical size of the cooling indices combining similar clusters of urban parks^9,24^. By comparing the cooling indices in the cooling volume bundles, the dominant cooling indices that exert cooling effects in different cooling volume bundles can be determined. In the hierarchical clustering method, the clustering output can be any number. Based on previous studies, 2–10 clusters were calculated and then analyzed by ANOVA test respectively^24^. The clustering scheme that passed the ANOVA test and had the least number of cluster groups was finally selected. The final clustering scheme in this study was divided into 4 groups, and the results of the ANOVA test are shown in Table S1.

### Identification of the TVoE

**Figure 4:**
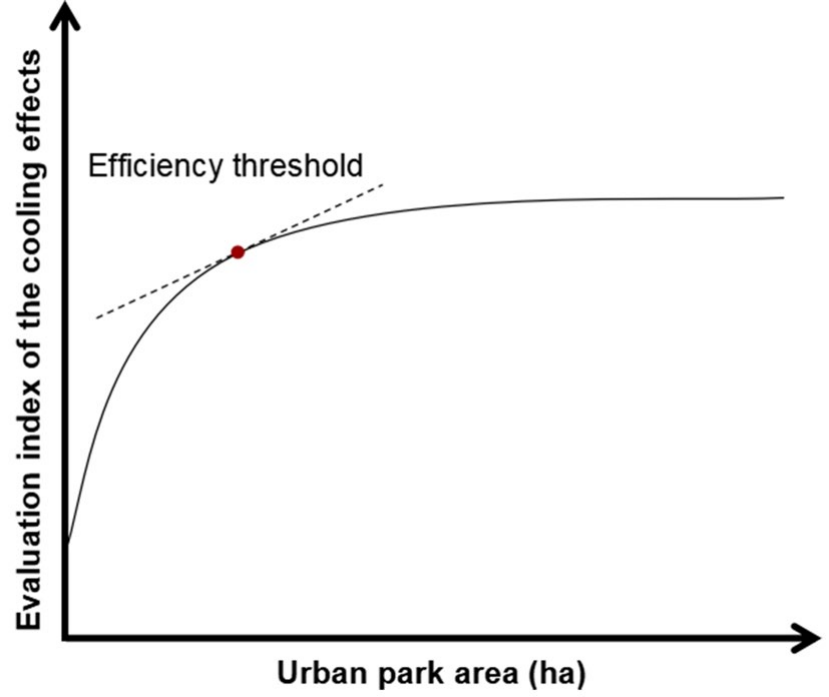
The sketch map of TVoE of the urban park.

Related studies have shown that the relationship between the influencing factors and the cooling effect is better fitted in a nonlinear curve^23,30^. Based on previous studies, the relationship between the cooling effect and park size was determined using the classical parametric logistic regression function (y = alnx + b)^17,24^. From a cost-effectiveness point of view, TVoE is the minimum area required for patches of green space, water bodies, and parks to achieve the optimal cooling effect. This concept focuses on the patch size when the maximum cooling effect is reached. When the size of a patch exceeds the TVoE, its cooling effect decreases or does not increase significantly. When the best-fit curve of the cooling effect to park size is logarithmic, TVoE is the patch size corresponding to when the slope of the fitted logarithmic curve is one^39,45^. In particular, changing the park size can affect its cooling effect, but it cannot expand the cooling effect indefinitely. When the park area changes to a certain value, the rate of change of the cooling effect slows down, which is the TVoE of the park area (Fig. 4).

### Assessment of accessibility and equity of cooling range

The accessibility of the cooling range of an urban park is the spatial accessibility of residents to the boundary of the park's cooling area. OD (origin destination) cost is crucial for evaluating the accessibility of urban parks, usually expressed in terms of travel time or distance between communities and parks^46^. We used minimum cost analysis to obtain minimum time–cost transportation data between residents and PCA through ArcGIS. An OD cost matrix model was constructed, and then the travel time costs in this model were evaluated for accessibility. Preprocessed the 2021 community data obtained from the Anjuke website, and finally obtained 2368 community data in the central urban area of Harbin. Then assume that each city park has an entrance at 200m intervals along the PCA boundary^17^. We calculated the time cost for people in each community to reach the nearest PCA boundary entrance by both walking and public transportation modes. The average walking speed was set to 1 m/s, and the waiting time at each intersection was set to 30 s^16^. The public transportation speed was set to 30 km/h, the stopping time at stations was set to 15 s, and the average signal waiting time at intersections was set to 30 s. According to the "Spatial Planning Guidelines: Community Living Units", published by the Ministry of Natural Resources of the People's Republic of China in 2021, the pedestrian accessibility and public transportation accessibility are categorized into 4 levels: within 5 min (very good), 5–10 min (good), 10–15 min (common), and more than 15 min (poor).

The Gini coefficient is a common indicator used in economics to measure income inequality among residents. It has been shown that the equity of urban park accessibility can be measured by the calculation of Lorenz curve graph with Gini coefficient^47^. We chose the neighborhoods (103 in total) in the central city of Harbin as the spatial unit of analysis. The population of the spatial units was calculated as the total number of households * household ratio (value = 2.23), based on the person-to-household ratio from the seventh census data published by the Harbin city government in 2021. The Gini coefficient was calculated using the formula:

6 $$G=1-\sum_{k=1}^{n}\left({P}_{k}-{P}_{k-1}\right)\left({R}_{k}+{R}_{k-1}\right)$$

where G is the Gini coefficient with a value range of [0, 1], ${\text{P}}_{\text{k}}$ is the cumulative proportion of the population within the spatial unit, and ${\text{R}}_{\text{k}}$ is the cumulative proportion of the time cost of the population within the spatial unit to reach the nearest PCA boundary entrance. If G = 0, it means that the urban park cooling range accessibility is completely equal. If G = 1, it means that urban park cooling range accessibility is not equal at all. The degree of equity can be determined from the relationship between the Gini coefficient and the degree of distributional homogeneity as specified by the United Nations Development Program (Table S2).

### Statistic analysis

In this study, the influencing factors were used as independent variables and the cooling indicators were used as dependent variables. Correlation analysis was used to explore the relationship between the influencing factors and the cooling indicators, and VIF (Variance Inflation Factor) was used to test the multicollinearity between the influencing factors. Because of the strong correlation between independent variables, ridge regression analysis is a regression analysis method used to deal with the problem of multicollinearity, so we used ridge regression to fit the equation^48^. In order to minimize the effect of data range, we logarithmically process the data before ridge regression, which can effectively improve the performance of the ridge regression model^49^. Dominant factors were identified based on regression analysis to quantify the relative contribution of each independent variable to the overall explanation of changes in cooling metrics^50^.

## Results

### Park cooling effect

**Figure 5:**
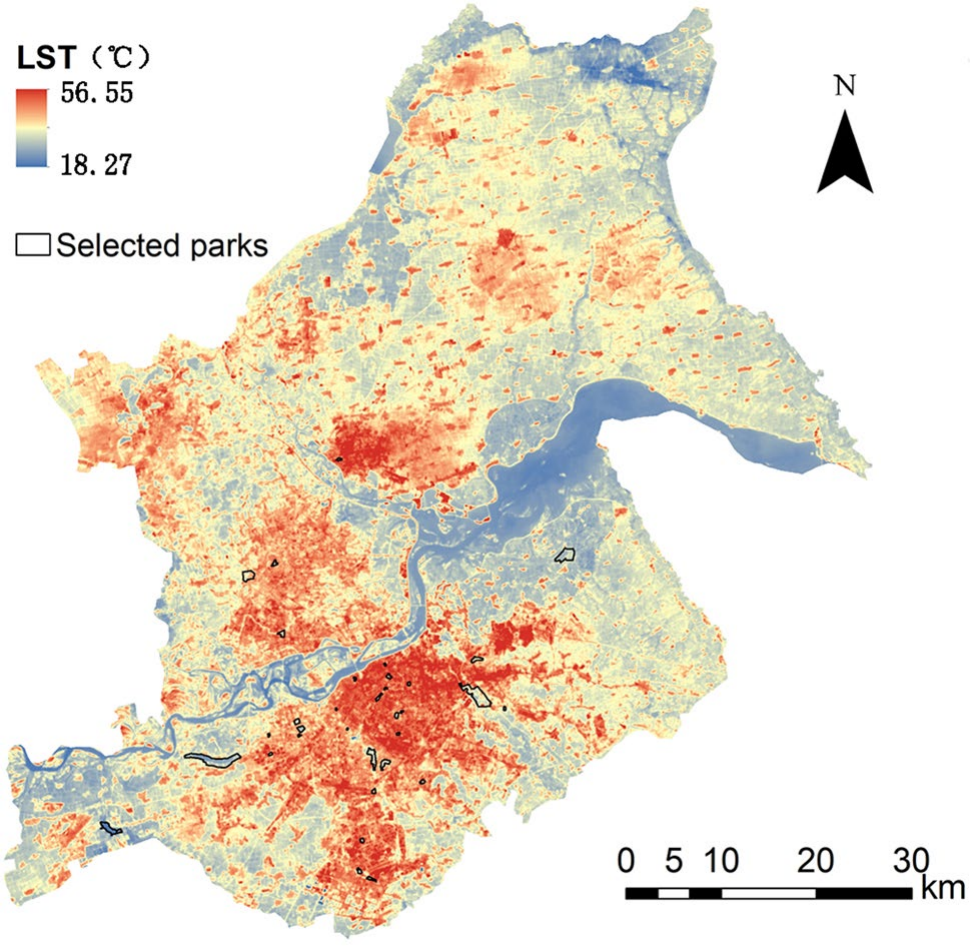
Averaged LST of the study area. Created using ArcGIS software and Adobe Illustrator. The base map was from the standard map with review number GS (2020) 4619 downloaded from the website of Standard Map Service of the Ministry of Natural Resources, with no modification of the base map (http://bzdt.ch.mnr.gov.cn/).

As shown in Fig. 5, the average LST of Harbin City in summer was 33.55 ℃, with an LST range interval of 18.27–56.55 ℃. From the spatial pattern of LST, all seven administrative districts of Harbin City had different degrees of UHI effects. The LST decreased gradually from southeast to northwest. The high-temperature zones were mainly located in the commercial core and dense residential areas in the southeast. The lower temperature zones were mainly found in large water areas and scattered city parks.

**Figure 6:**
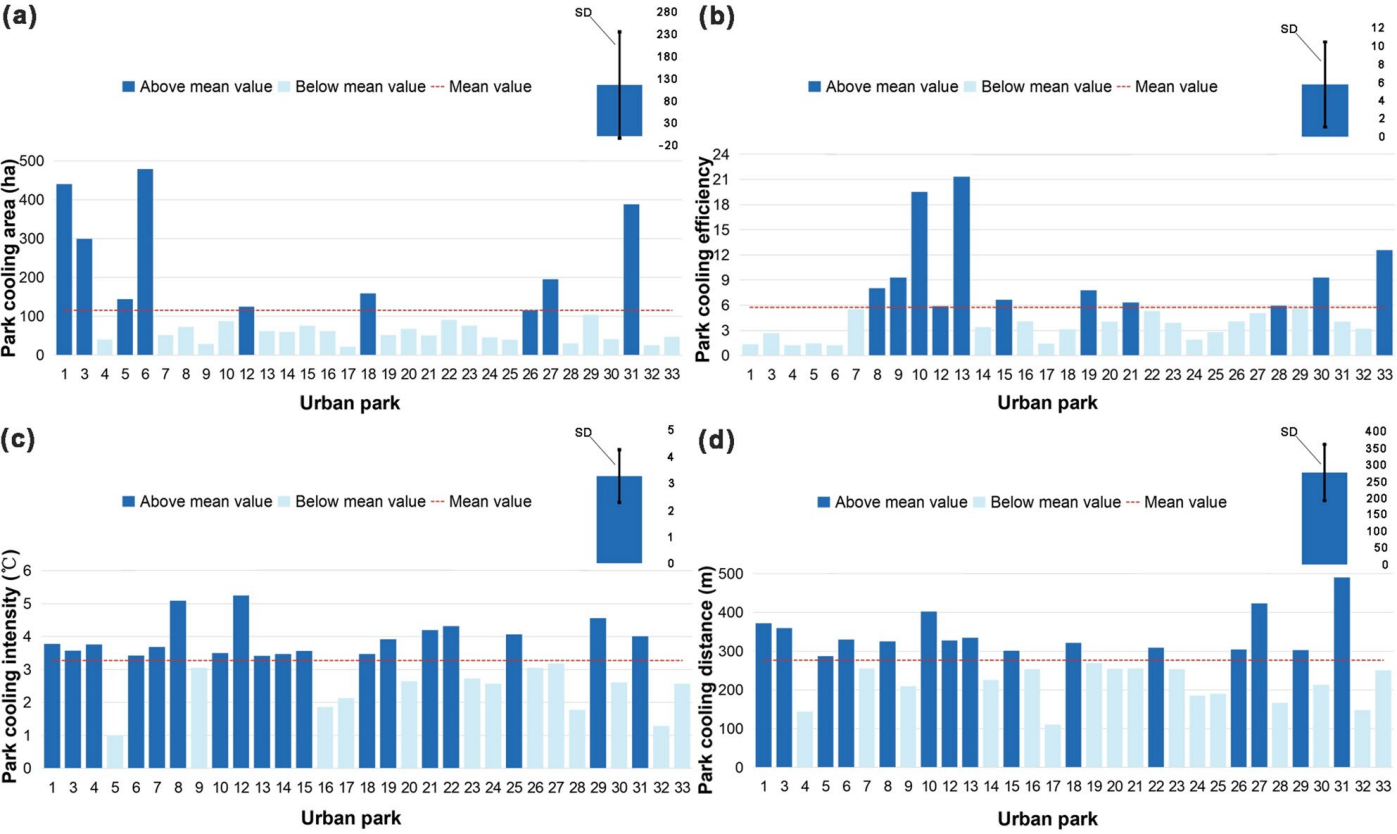
Four cooling indices for 31 city parks (only those with cooling effects. N = 31, error bars indicate the standard deviation. *SD*  standard deviation). (**a**) PCA index for 31 urban parks, (**b**) PCE index for 31 urban parks, (**c**) PCI index for 31 urban parks, (**d**) PCD index for 31 urban parks.

**Figure 7:**
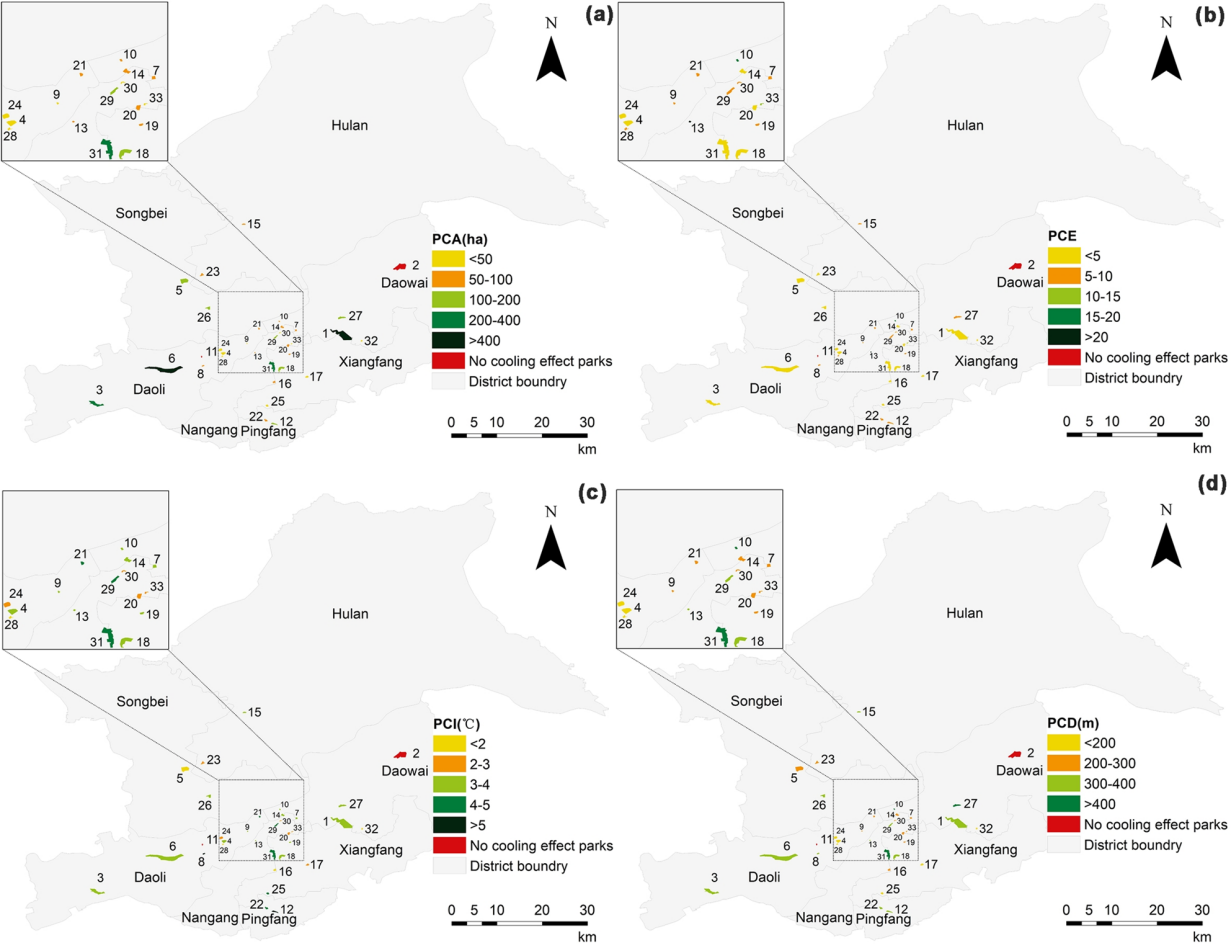
Spatial distribution of the four cooling indicators. (**a**) PCA distribution of city parks, (**b**) PCE distribution of city parks, (**c**) PCI distribution of city parks, (**d**) PCD distribution of city parks.

Of the 33 urban parks, 31 parks were cooler than the surrounding area, while 2 parks had no cooling effect. Park 30 had the highest internal LST of 33.16 ℃, and Park 3 had the lowest internal LST of 24.79 ℃, with a difference of about 8 ℃. Figure 6 shows the results of PCA, PCE, PCI, and PCD for the 31 parks (only those with cooling effects). The mean PCA value for urban parks with cooling effects was 115.35 ha, ranging from 21.80 to 479.36 ha, with Park 6 having the highest PCA (Fig. 6a). The mean PCE value was 5.74, ranging from 1.25 to 21.35, with Park 13 having the highest PCE (Fig. 6b). The mean PCI value was 3.27 ℃, ranging from 1.01 to 5.24 ℃, with Park 12 having the highest PCI (Fig. 6c). The mean value of PCD was 277 m with a range of 110–490 m, with Park 31 having the highest PCD (Fig. 6d). The spatial distribution maps of the four cooling indicators showed that parks with larger PCA were mainly located in the suburban areas of the city, which generally have lower building and population densities (Fig. 7a). In contrast to PCA, parks with higher PCE were mainly located in the center of the city (Fig. 7b). Parks with larger PCI were mainly located in the commercial core and dense residential areas in the southeast (Fig. 7c). Parks with larger PCD were mostly located in the suburbs of the city (Fig. 7d).

### Influencing factors of park cooling effect

Regression analysis was used to identify the dominant factors influencing the cooling effect and to quantify the relative contribution of each independent variable to the overall explanation of the change in the cooling indicator. The regression analysis results of PCI, PCD, PCA, and PCE are shown in Table S3. All retention factors (independent variable) were significant at P < 0.1. The F-values of the model were 2.235, 4.24, 15.378, and 7.603 with p (level of significance) < 0.05, which proved that the simulation was better. The influences with the highest standardized regression coefficients were identified as dominant factors^51^.

It can be seen that Grey contributed 50% to PCI. NDVIin, Blue, Greenout, TR and Grey contributed 76% to PCD. Area, Perimeter, LSI, Greenin, Blue, Greenout, TR, SH and Grey contributed 92% to PCA. Area, Perimeter, Greenin, Greenout, TR and Grey contributed 85% to PCE. The relative contribution of Blue to PCD among the internal factors was the largest at 16%. The relative contribution of Grey to PCD among the external factors was the largest at 31%. The relative contribution of Blue to PCA among the internal factors was the largest at 12%. The relative contribution of Grey to PCA among the external factors was the largest at 19%. The area had the largest relative contribution to PCE among the internal factors at 28%. The relative contribution of Grey to PCE was the largest among the external factors at 20%.

### Identification of different cooling bundles of parks

**Figure 8:**
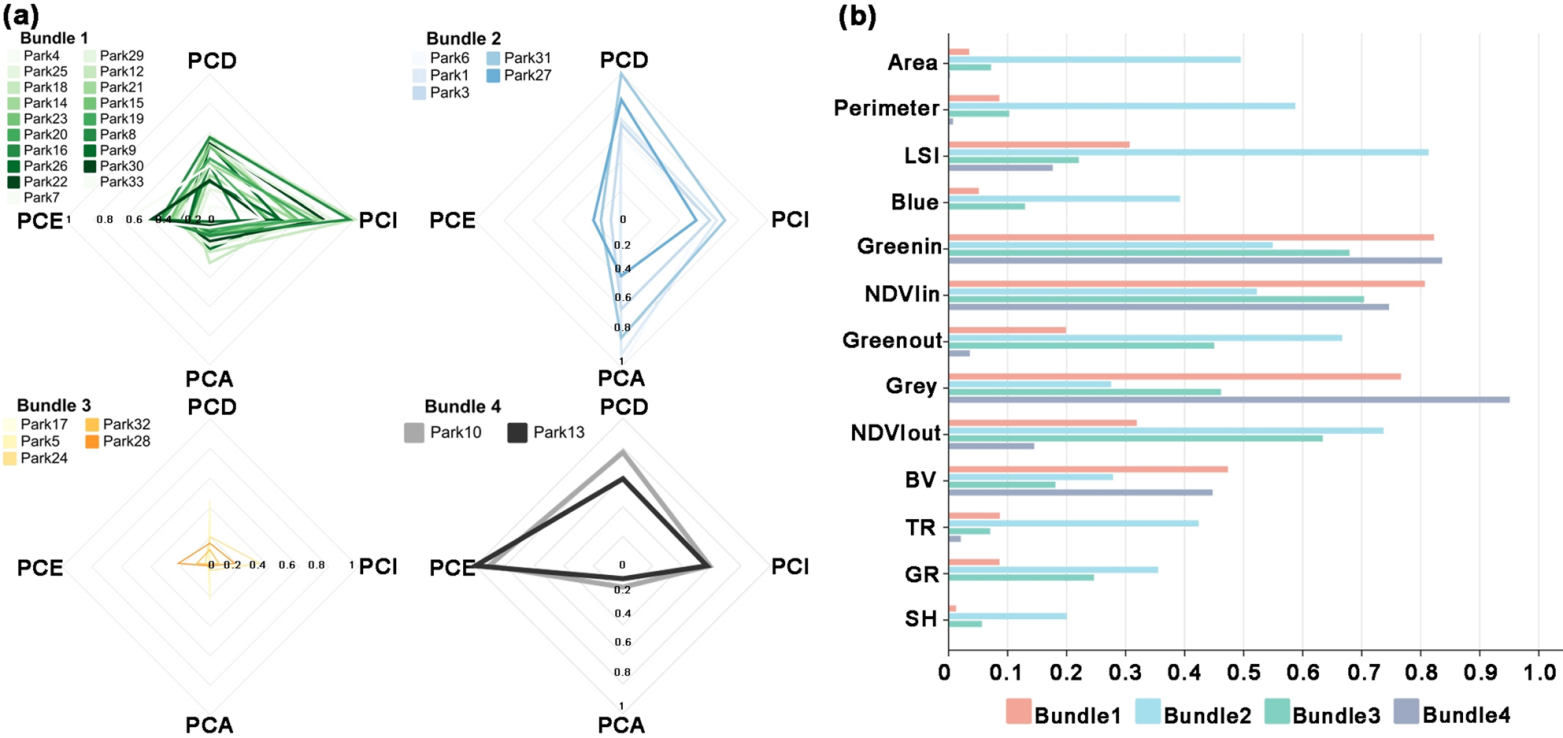
Comparison of four park cooling capacity bundles. (**a**) Radar charts of cooling indicators of four park cooling capacity bundles, (**b**) mean values of influencing factors of four park cooling capacity bundles.

Applying the hierarchical clustering method, this study classified the parks with cooling effects into four categories. According to Peng's definition, these four categories are called cooling bundles 1, 2, 3, and 4 (Fig. 8a)^9^. Then, the mean values of the influencing factors in each cooling bundle were counted (Fig. 8b), and the differences among the four cooling bundles were further analyzed. The results showed the following:

Cooling Bundle 1: performs well in terms of PCI. Compared to the other three cooling indices, this type of park had a larger PCI and significantly reduced the ambient temperature of its surroundings, with an average PCI of 3.57 °C. These parks had the largest NDVIin and BV.

Cooling Bundle 2: parks dominated by cooling area and cooling distance. The PCA and PCD of this park type were larger compared to other cooling indices. The mean PCA was 360.43 ha and the mean PCD was 395 m. This group of parks was characterized by the largest area, perimeter, LSI, Blue, TR, GR, SH, Greenout, and NDVIout, and the smallest Greenin, NDVIin, and Grey.

Cooling Bundle 3: all four cooling indices are low for this park type. This group of parks was characterized by the smallest values of LSI and BV.

Cooling Bundle 4: cooling efficiency dominated parks. This type of park had a larger PCE compared to the other three cooling indices, with an average PCE of 20.42. This group of parks had the largest Greenin and Grey, but the smallest park area.

### Determination of TVoE of park area

**Figure 9:**
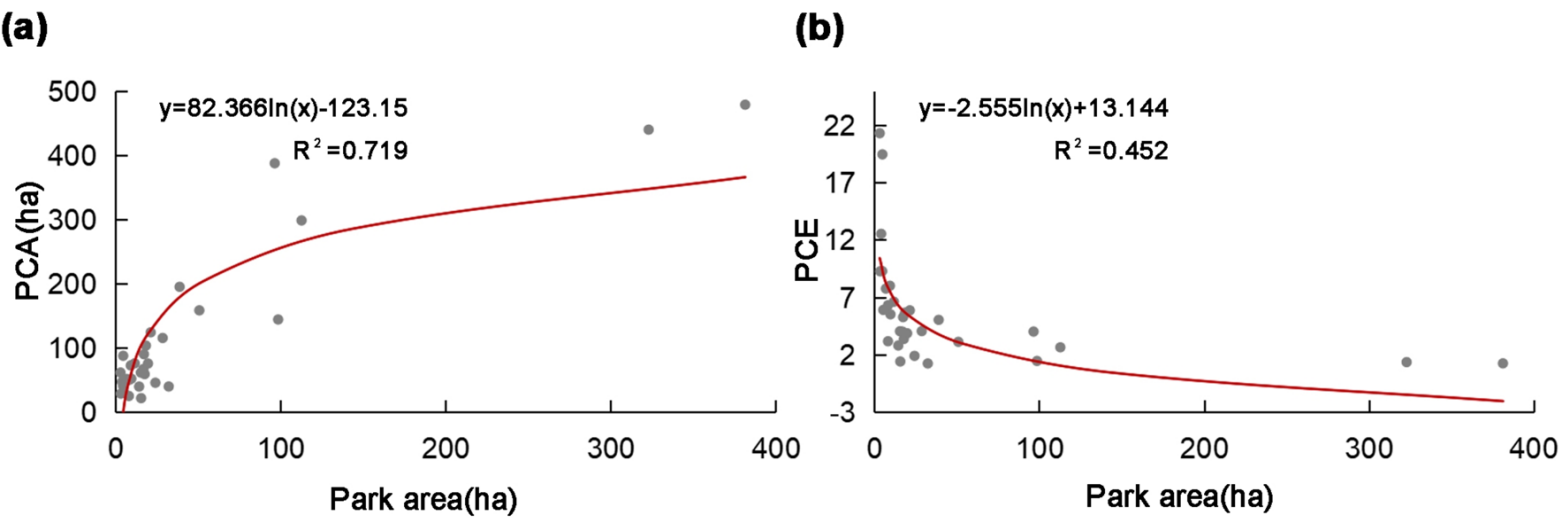
TVoE for park areas with different cooling indices. (**a**) TVoE of park area for PCA, (**b**) TVoE of park area for PCE.

According to Table S3, it can be observed that the park area was an important factor for PCA and PCE. Therefore, the relationship between PCA, PCE, and park area was further determined using the classical parametric logistic regression function (y = alnx + b). As shown in the equation in Fig. 9a, y = 82.366ln(x) − 123.15 (R^2^ = 0.719), the independent variable (x) was "park area", and the dependent variable (Y) was the cooling effect indicator "PCA". When the slope of the logarithmic function was 1, the rate of change of the fitted curve of PCA decreased, and the TVoE of the park area was 82.37 ha. As shown in the equation in Fig. 9b, y = −2.555ln(x) + 13.144 (R^2^ = 0.452), the independent variable (x) was the "park area", and the dependent variable (Y) was the cooling effect indicator "PCE". When the park area increased to 2.56 ha, the slope of the logarithmic function was 1, and the rate of change of the fitted curve of PCE decreased, which was the efficiency threshold of the park area. It can be found that the TVoE of the park area calculated based on different cooling effect evaluation indices varies significantly. In summary, the TVoE of the urban park in this study based on PCA was 82.37 ha, and the TVoE based on PCE was 2.56 ha. The appropriate cooling effect evaluation index should be considered to calculate the corresponding TVoE in the actual project, from which targeted planning and design can be made.

### Accessibility and equity analysis of cooling range

Tables 2 and 3 showed the statistical results for the percentage of communities with different travel times under walking and public transportation modes, respectively. The percentage of communities with access to park cooling ranges within 15 min of walking and public transportation were 39.2% and 94.01%, respectively. 10.05% of communities were located within the cooling range and can directly enjoy cooling services in urban parks. By walking for 5 min, 5–10 min, and 10–15 min, 7.47%, 10.58%, and 11.11% of communities can enjoy park cooling services, respectively. 60.8% of communities were unable to enjoy the cooling effect of these parks within a 15 min walk. By public transportation for 5 min, 5-10 min, and 10–15 min, 35.6%, 35.6%, and 12.77% of communities can enjoy park cooling services, respectively. 5.99% of communities were unable to enjoy the cooling effect of these parks within 15 min of public transportation.

**Table 2:** Statistics on the percentage of communities with different time consumption in walking mode.

Time consumption (min)	Within PCA	< 5 min	5–10 min	10–15 min	> 15 min
Inside the 2st ring road (%)	10.11	7.96	12.54	12.76	56.63
Between the 2st and 3nd ring roads (%)	11.71	7.29	9.06	9.28	62.65
Outside the 3nd ring road (%)	5.33	5.92	6.51	9.17	73.08
Total (%)	10.05	7.47	10.58	11.11	60.8

**Table 3:** Statistics on the percentage of communities with different time consumption in public transport mode.

Time consumption (min)	Within PCA	< 5 min	5–10 min	10–15 min	> 15 min
Inside the 2st ring road (%)	10.11	38.85	39.35	11.47	0.22
Between the 2st and 3nd ring roads (%)	11.71	33.15	31.6	14.36	9.17
Outside the 3nd ring road (%)	5.33	28.7	30.77	13.91	21.3
Total (%)	10.05	35.6	35.6	12.77	5.99

The Gini coefficient results for walking and public transportation calculated based on population data and accessibility time cost data were shown in Table S4. The research results showed the degree of equality among residents in each neighborhood in reaching the cooling range of the park under two transportation modes. The Gini coefficient results for most neighborhoods were generally consistent across the two modes of transportation. We calculated the average of the Gini coefficients of the two transportation modes for each neighborhood. The equity of cooling range accessibility for the 103 neighborhoods was shown in Table 4. It can be found that out of 103 neighborhoods, 52 neighborhoods were at an absolute equality level, 15 neighborhoods were at a comparative equality level, 18 neighborhoods were at a relative reasonableness level, 15 neighborhoods were at a large disparity level, and 3 neighborhoods were at a wide disparity level.

**Table 4:** Distribution intervals for equity of cooling range accessibility in 103 neighborhoods.

Average Gini coefficient	Number of neighborhoods
0–0.2	52
0.2–0.3	15
0.3–0.4	18
0.4–0.6	15
0.6–1	3

## Discussion

### LST, park cooling effect, and influencing factors

**Figure 10:**
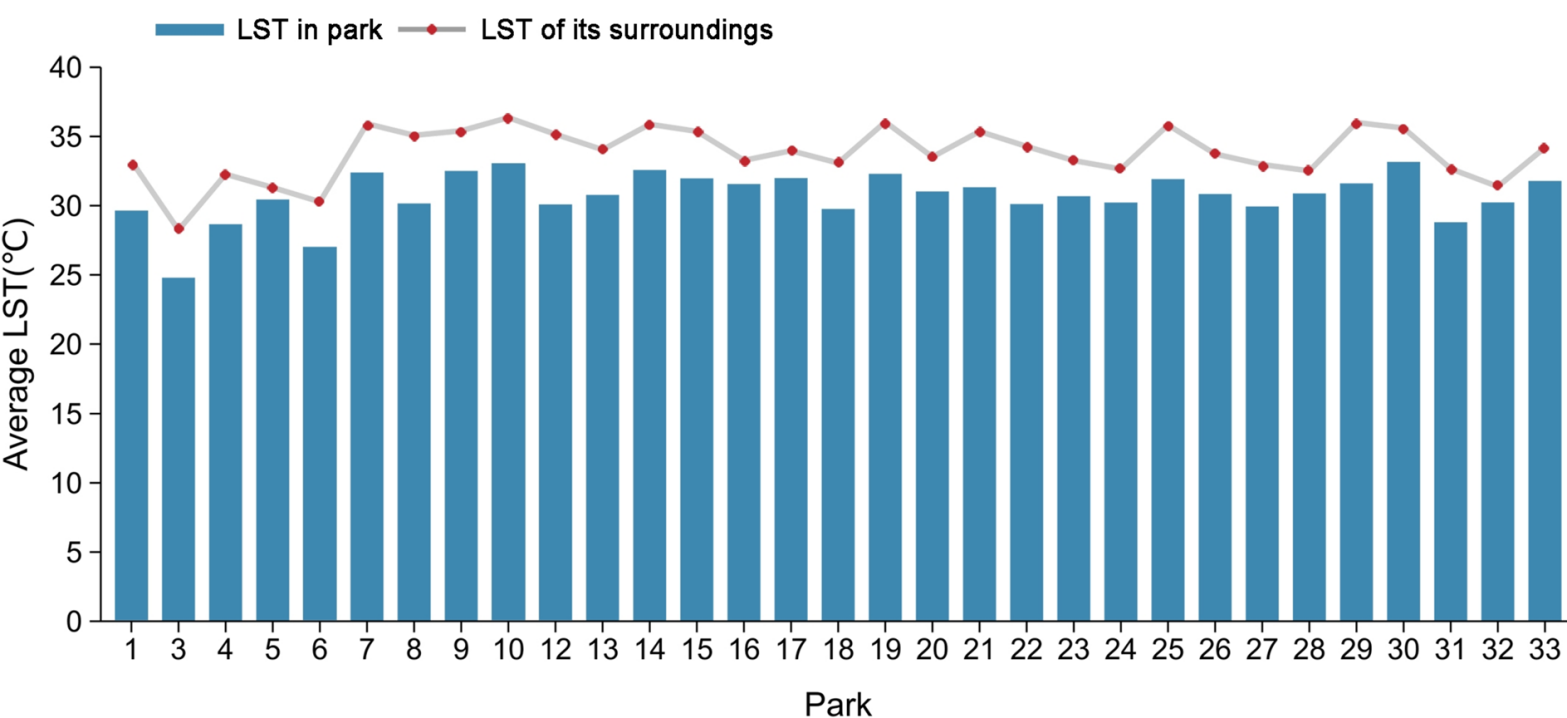
Average LST and surrounding temperature of 31 parks (only those with cooling effects).

Of the 33 urban parks, 31 were cooler than the surrounding area. It shows that most of the urban parks have a cooling effect and can effectively mitigate the UHI effect. We compared the 31-park interior LST with a cooling effect with the surrounding environment LST (the LST of the first turning point). As shown in Fig. 10, the LST of the park's surroundings was significantly higher than the LST of the park's interior, with a difference of 3.27 °C between the two. We also found significant differences in internal LST among different parks, with a maximum difference of about 8 °C. This indicates that the differences in landscape composition and surrounding environment of urban parks can significantly affect the cooling effect of the park. In addition, two parks in our study had no cooling effect (Parks 2 and 11). Comparing the results of related studies in other cities, not all parks had a cooling function^15,16^. It was found that the climatic background factor is not the cause of this phenomenon but caused by the park's characteristics and the surrounding complex landscape environment.

In this study, the average PCA was 115.35 ha in Harbin. Compared the findings with other cities such as Shanghai (45.82 ha), Shenzhen (52.35 ha), Nanchang (44.49 ha), Hohhot (33.97 ha), and Jiuquan (28.32 ha)^52^. The cooling effect of parks in Harbin City in the severe cold region was characterized by a larger PCA. The average PCE was 5.74 in Harbin. Comparing the results of the studies in Shenzhen (1.36), Wuhan (4.5), and Fuzhou (2.77)^9,16,24^. The PCE was also larger in Harbin City Park. The climatic background of different cities may contribute to the differences^16,52^.

Another study of the cooling effect of green spaces in Harbin found that area and perimeter were the main factors influencing the cooling effect^35^. The results of this paper verified this conclusion, and we also calculated the contribution of these factors to the cooling effect. The relative contributions of area and perimeter to PCA were 8% and 11%, respectively. The relative contributions of area and perimeter to PCE were 28% and 18%, respectively. In addition, previous studies have neglected the influence of internal water body factors and external factors on the cooling effect. In this study, we found that parks with higher internal water body area had larger PCD and PCA (relative contributions of 16% and 12%, respectively). Grey had the greatest effect on PCI, PCD, PCA and PCE among the park external factors. The relative contributions were 100%, 31%, 19% and 20%, respectively. It indicates that the building density around the park can significantly affect the cooling effect of the park. However, the BV had no significant effect on the cooling effect. The possible reason is that compared to building volume, building density has a stronger relationship with factors such as spatial distribution and ventilation. Therefore, building density has a greater impact on the cooling effect. For vegetation types, we found that increasing the area of TR and SH in the park can increase the PCD and PCA of the park. GR has no effect on the four indicators of the park. It indicates that the selection of trees and shrubs planting is more effective than grass for increasing the cooling effect of the park. The above indicates that in the planning and design of parks in Harbin City, we should not only pay attention to the area and perimeter of the parks, but the construction of water bodies inside the parks, the selection of vegetation types and the density of buildings around the parks also have a greater impact on the cooling effect.

### Implications for the design of urban parks

#### Cooling capacity bundle

Urban parks are an important way to improve the urban environment. Previous studies using a single indicator to quantify the cooling effect of urban parks are inappropriate^53^. The characteristics of the parks and the complex environmental factors around them can affect the cooling effect of the parks. Because different cooling indicators are affected by different factors, this study used four cooling indices to quantify the different cooling effects of urban parks from multiple perspectives. The parks were categorized into different cooling capacity bundles based on the four indices. In cooling capacity bundle 1, PCI performed well, but the water body area was low. When renovating and designing this group of parks, some water landscape construction should be added. From cooling capacity bundle 2, it can be seen that they had larger PCA and PCD. This group of parks had less green space inside and poor vegetation growth. It is possible that these parks had insufficient financial investment, resulting in a lack of green infrastructure. In the future, these types of parks should focus on enhancing their internal green spaces. The overall cooling capacity of the park in cooling capacity bundle 3 was poor, and there was a lot of room for renovation in future urban planning and landscape design. The cooling potential of these parks can be improved by expanding the park area and increasing the construction of internal blue-green infrastructure. As can be seen from cooling capacity bundle 4, the parks with better PCE were characterized by smaller areas and dense vegetation growth. However, the area of water bodies was low and some water landscaping should be added. These types of parks did not cover a large area and were characterized by low capital investment and high cooling efficiency. The construction should be promoted in highly urbanized and land-scarce areas in the future. The use of four cooling indices to classify parks into four groups based on different cooling characteristics can provide a full understanding of the current cooling situation of existing parks. These findings provide some reference for urban decision makers to carry out park renovation and improve the cooling capacity of parks.

#### Threshold area

Due to the scarcity of urban land resources, the threshold area of parks under the maximization of cooling effect needs to be addressed urgently. Most studies calculated threshold areas based on a single indicator that is incomplete. This study was based on PCA and PCE to obtain different threshold areas (82.37 ha and 2.56 ha, respectively), and decision-makers can choose corresponding solutions according to different cooling needs. For example, in highly urbanized commercial core areas where available space is very limited, urban decision-makers should choose TVoE (2.56 ha) based on PCE calculation. This will maximize the cooling efficiency with minimum area under the condition of very limited land resources. If the available land space is sufficient, such as in large-scale residential areas, our demand is to maximize the cooling area of parks, in which case the city decision maker should choose the TVoE based on PCA calculation (82.37 ha). This will allow more residents to enjoy the cooling services of the park. In summary, our advantage is that urban planners can choose different threshold areas according to different cooling needs.

### Accessibility and equity of cooling range in urban parks

#### Accessibility

**Figure 11:**
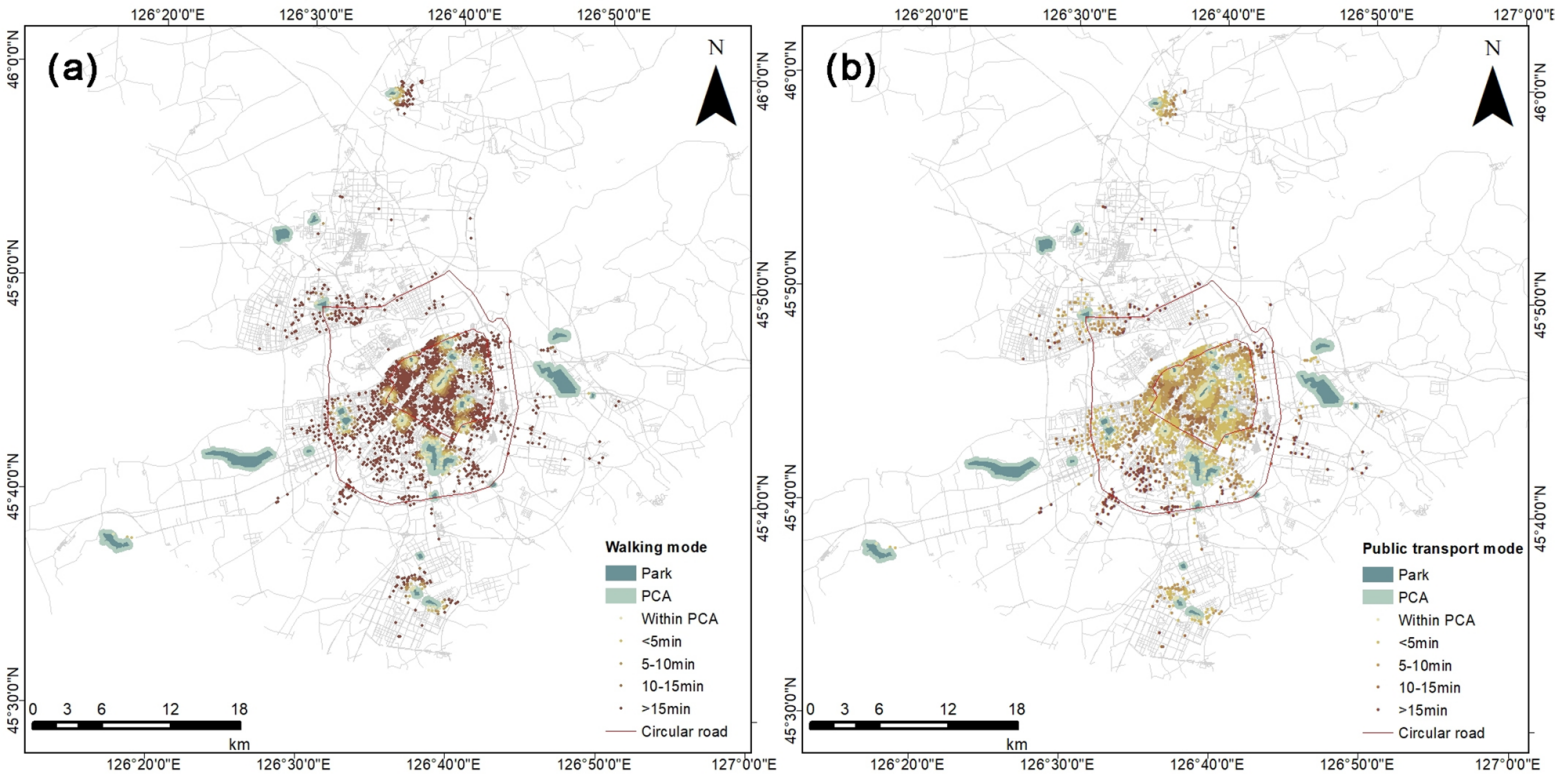
Spatial distribution of community cooling range accessibility for different transportation modes and different travel times. (**a**) Walking accessibility, (**b**) public transportation accessibility.

With the rapid development of the economy, people's demand for a better living environment is growing. The benefits of parks as a public resource of society are getting more and more attention. Similar to income inequality, the uneven distribution of urban parks can affect people's frequency of use, which in turn prevents them from better enjoying the benefits of parks. While most previous studies focused on the cooling effect of parks, this study not only explored the optimal park area that maximizes the cooling effect of multiple indicators, but also evaluated the accessibility and equity of existing urban parks by combining analytical tools such as Arcgis and the Gini coefficient. Figure 11 showed the spatial distribution characteristics of communities with different travel times under different transportation modes. It can be found that the community had very poor accessibility to walking transportation modes in comparison to public transportation modes. In walking mode, 56.63%, 62.65%, and 73.08% of communities within ring 2, between ring 2–3, and outside ring 3 cannot reach the cooling range of the park within 15 min, respectively. In public transportation mode, 0.22%, 9.17%, and 21.3% of communities within ring 2, between ring 2–3, and outside ring 3 cannot reach the cooling range of the park within 15 min, respectively. It can be found that the accessibility for residents to enjoy park cooling services became worse as the ring road spreads outward. In addition, from the distribution of parks and communities outside the third ring road, it can be seen that there were more large parks outside the third ring road, with a lower population density and a more dispersed population distribution. This results in a minority of people enjoying most of the space resources, while others faced poor accessibility or even inability to enjoy cooling services. These illustrated the inadequate supply of parks and the inequitable distribution of parks in the central urban area of Harbin.

#### Equity

**Figure 12:**
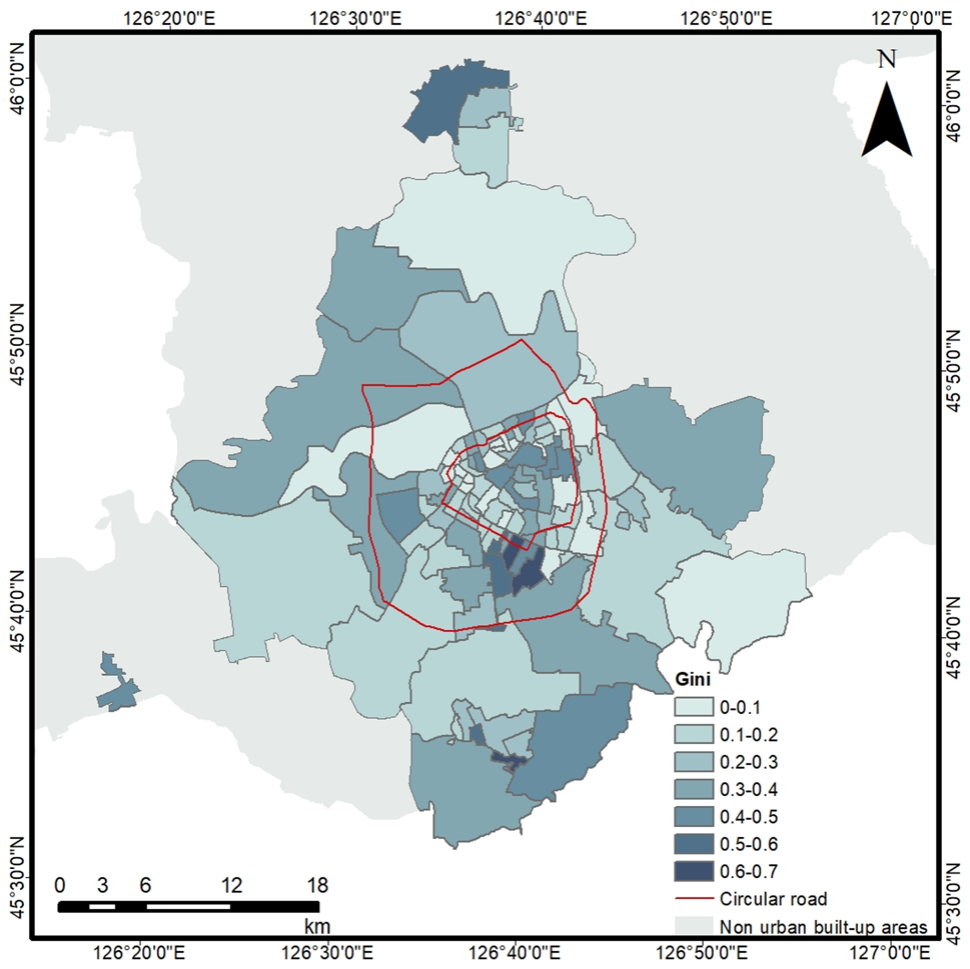
Spatial distribution of equality differences in park cooling services enjoyed by residents in 103 neighborhoods (average of two transportation modes). Created using ArcGIS software. Neighborhood administrative boundaries were from BIGEMAP GIS Office software (http://www.bigemap.com/).

It has been shown that parks should be planned at the scale of neighborhoods as the demand for summer cooling by urban residents continues to increase^16^. Previous studies have rarely evaluated the equality of residents in enjoying park cooling services on a neighborhood scale. This study evaluated the equality of the cooling range of 103 neighborhoods in the central urban area under walking and public transportation modes, using the neighborhood as a spatial unit (Figure S1). We found that the level of equality in the same neighborhood was essentially the same for both modes of travel. The average of the Gini coefficients of the two modes of transportation was further selected for a comprehensive assessment, as shown in Fig. 12. It can be observed that there was a significant gap in equality between different neighborhoods (Gini = 0–0.69), indicating that the equitable distribution of park resources has become an urgent demand at present. We also found that nearly 18% of neighborhoods were experiencing severe inequality in cooling range accessibility (Gini > 0.4). More than 50% of the neighborhoods with absolute equality (Gini = 0–0.2) were located within the 2nd ring. As the ring road spread outward, the number of absolutely equal neighborhoods gradually decreased. Residents of the inner and outer ring roads do not enjoy equal cooling services.

Government investment in the construction of parks is limited, and there is a need for policymakers to understand the differences in equity in access to cooling services for all neighborhoods. Priority could be given to allocating park resources to neighborhoods with severe inequities in cooling services (Gini > 0.4). Our findings can help local governments and city planners more accurately allocate park resources based on the equality of neighborhood access to cooling services.

## Conclusions

Thirty-three urban parks in Harbin City were used as research objects. This study provided planning and suggestions for maximizing the cooling effect of urban parks and rational allocation of park resources. The cooling effect of urban parks was quantified, identifying the dominant factors influencing the cooling effect and their relative contribution rates. Due to the scarcity of urban land, it is urgent to determine the threshold for park area to maximize the cooling effect. Previous studies that calculated the TVoE of park size from a single perspective were not comprehensive. This study quantified the TVoE of park size based on both PCA and PCE perspectives. City decision makers can choose different threshold areas based on different cooling needs. An OD cost matrix was constructed to calculate the spatial accessibility of the community to the park cooling range. Using the Gini coefficient to assess the equity of access to cooling services for neighborhood unit residents. From the perspective of social equity, it can help local governments and planners allocate park resources more accurately based on the differences in equality between neighborhoods. The following conclusions were drawn:

- 31 out of 33 city parks had a significant cooling effect, they can reduce the average LST of the surrounding environment by 3.27℃. The average PCA was 115.35 ha, the average PCE was 5.74, the average PCI was 3.27℃, and the average PCD was 277 m. Compared to other cities, the cooling effect of parks in Harbin City in the severe cold region was characterized by larger PCA and PCE;
- The external gray space area was the dominant factor for PCI, PCD, and PCA (with relative contribution rates of 100%, 31%, and 19%, respectively). Park area was the dominant factor of PCE (with a relative contribution rate of 28%). The construction of water landscape can improve the PCD and PCA of the park. Trees and shrubs were more effective in improving the cooling effect of parks than grasslands;
- Urban parks were categorized into four cooling capacity bundles based on four cooling indices, and significant differences were found in the cooling effect of urban parks with different cooling capacity bundles. In utilizing the advantages and roles of different types of parks, parks with great cooling potentials should be enhanced. Provide a certain reference for the cooling capacity renovation of existing parks;
- Based on two indices, PCA and PCE, the TVoE of urban parks in Harbin City in the severe cold region were calculated as 82.37 ha and 2.56 ha, respectively. Urban planners can choose different solutions for different cooling needs. From a cost-effective point of view, the best cooling effect is achieved with the smallest park area;
- 60.8% and 5.99% of communities cannot reach the cooling range of the park within 15 min in walking mode and public transportation mode, respectively. Approximately 18% of neighborhood residents were experiencing severe inequities in cooling range accessibility. The accessibility and equality of residents enjoying park cooling services were gradually decreasing as they spread from the inner ring to the outer ring.

Our paper had certain limitations. First, 600 m was identified as the buffer range for this study, but this may not be appropriate for all parks given the unique environmental and climatic context of each park. Second, this study examines the cooling effect of urban parks using summer as the study time. Seasonal changes have an impact on the cooling effect and UHI effect in urban parks^7,54^. Conducting seasonal comparisons of park cooling effects is a priority for future research. In addition, land surface temperature data with higher resolution can be used in the future to improve the accuracy of the study results. For example, drones using high-performance thermal imaging cameras to obtain higher land surface temperature data. Finally, in future research we should consider the differences in the demand for cooling services among different populations, such as the differences between the elderly, children and young people. In the background of global warming and accelerated urbanization, land resources are scarce in cities, and it is crucial to maximize the cooling effect of urban parks as well as the rational allocation of park resources. Local governments and urban planners should prioritize more equitable cooling services for vulnerable groups.

### Supplementary Information

Supplementary Information.

## Data Availability

The data that support the findings of this study are available on request from the corresponding author upon reasonable request.

## References

[1] Zhou W, Cao W, Wu T, Zhang T (2023). The win–win interaction between integrated blue and green space on urban cooling. Sci. Total Environ..

[2] Ali G (2021). Environmental impacts of shifts in energy, emissions, and urban heat island during the COVID-19 lockdown across Pakistan. J. Clean Prod..

[3] Yao L, Li T, Xu MX, Xu Y (2020). How the landscape features of urban green space impact seasonal land surface temperatures at a city-block-scale: An urban heat island study in Beijing, China. Urban For. Urban Green..

[4] Liaqat W (2022). Climate change in relation to agriculture: A review. Span. J. Agric. Res..

[5] Akbari H, Kolokotsa D (2016). Three decades of urban heat islands and mitigation technologies research. Energy Build..

[6] Harmay NSM, Kim D, Choi M (2021). Urban Heat Island associated with Land Use/Land Cover and climate variations in Melbourne, Australia. Sust. Cities Soc..

[7] Lin PY, Lau SSY, Qin H, Gou ZH (2017). Effects of urban planning indicators on urban heat island: A case study of pocket parks in high-rise high-density environment. Landsc. Urban Plan..

[8] Kim Y, Yu SY, Li DY, Gatson SN, Brown RD (2022). Linking landscape spatial heterogeneity to urban heat island and outdoor human thermal comfort in Tokyo: Application of the outdoor thermal comfort index. Sust. Cities Soc..

[9] Peng J (2021). How to quantify the cooling effect of urban parks? Linking maximum and accumulation perspectives. Remote Sens. Environ..

[10] Yu ZW (2020). Critical review on the cooling effect of urban blue-green space: A threshold-size perspective. Urban For. Urban Green..

[11] García-Haro A, Arellano B, Roca J (2023). Quantifying the influence of design and location on the cool island effect of the urban parks of Barcelona. J. Appl. Remote Sens..

[12] Yu ZW, Guo XY, Jorgensen G, Vejre H (2017). How can urban green spaces be planned for climate adaptation in subtropical cities?. Ecol. Indic..

[13] Gao Z, Zaitchik BF, Hou Y, Chen WP (2022). Toward park design optimization to mitigate the urban heat Island: Assessment of the cooling effect in five US cities. Sust. Cities Soc..

[14] Feyisa GL, Dons K, Meilby H (2014). Efficiency of parks in mitigating urban heat island effect: An example from Addis Ababa. Landsc. Urban Plan..

[15] Qiu KB, Jia BQ (2020). The roles of landscape both inside the park and the surroundings in park cooling effect. Sust. Cities Soc..

[16] Chen M, Jia WX, Yan L, Du CL, Wang K (2022). Quantification and mapping cooling effect and its accessibility of urban parks in an extreme heat event in a megacity. J. Clean Prod..

[17] Shi MQ, Chen M, Jia WX, Du CL, Wang YT (2023). Cooling effect and cooling accessibility of urban parks during hot summers in China’s largest sustainability experiment. Sust. Cities Soc..

[18] Geng XL (2022). The influence of local background climate on the dominant factors and threshold-size of the cooling effect of urban parks. Sci. Total Environ..

[19] Zhou W, Yu WL, Wu T (2022). An alternative method of developing landscape strategies for urban cooling: A threshold-based perspective. Landsc. Urban Plan..

[20] Jaganmohan M, Knapp S, Buchmann CM, Schwarz N (2016). The bigger, the better? The influence of urban green space design on cooling effects for residential areas. J. Environ. Qual..

[21] Monteiro MV, Doick KJ, Handley P, Peace A (2016). The impact of greenspace size on the extent of local nocturnal air temperature cooling in London. Urban For. Urban Green..

[22] Feng XJ (2023). Quantifying and comparing the cooling effects of three different morphologies of urban parks in Chengdu. Land.

[23] Chang CR, Li MH, Chang SD (2007). A preliminary study on the local cool-island intensity of Taipei city parks. Landsc. Urban Plan..

[24] Yao X (2022). How can urban parks be planned to mitigate urban heat island effect in "Furnace cities" ? An accumulation perspective. J. Clean Prod..

[25] Du CL, Jia WX, Chen M, Yan L, Wang K (2022). How can urban parks be planned to maximize cooling effect in hot extremes? Linking maximum and accumulative perspectives. J. Environ. Manag..

[26] Duncan JMA (2019). Turning down the heat: An enhanced understanding of the relationship between urban vegetation and surface temperature at the city scale. Sci. Total Environ..

[27] Cao X, Onishi A, Chen J, Imura H (2010). Quantifying the cool island intensity of urban parks using ASTER and IKONOS data. Landsc. Urban Plan..

[28] Xie QJ, Li J (2021). Detecting the cool island effect of urban parks in Wuhan: A city on rivers. Int. J. Environ. Res. Public Health.

[29] Xiao Y (2023). A comprehensive framework of cooling effect-accessibility-urban development to assessing and planning park cooling services. Sust. Cities Soc..

[30] Brown G, Rhodes J, Dade M (2018). An evaluation of participatory mapping methods to assess urban park benefits. Landsc. Urban Plan..

[31] Yan H, Wu F, Dong L (2018). Influence of a large urban park on the local urban thermal environment. Sci. Total Environ..

[32] Yang JR, Guo R, Li D, Wang XL, Li FZ (2022). Interval-thresholding effect of cooling and recreational services of urban parks in metropolises. Sust. Cities Soc..

[33] Yang CB (2017). The effect of urban green spaces on the urban thermal environment and its seasonal variations. Forests.

[34] Yang CB (2017). The cooling effect of urban parks and its monthly variations in a snow climate city. Remote Sens..

[35] Huang M, Cui P, He X (2018). Study of the cooling effects of urban green space in Harbin in terms of reducing the heat island effect. Sustainability.

[36] Tan XY, Sun X, Huang CD, Yuan Y, Hou DL (2021). Comparison of cooling effect between green space and water body. Sust. Cities Soc..

[37] Jimenez-Munoz JC, Sobrino JA, Skokovic D, Mattar C, Cristobal J (2014). Land surface temperature retrieval methods from Landsat-8 thermal infrared sensor data. IEEE Geosci. Remote Sens. Lett..

[38] Yang GY, Yu ZW, Jorgensen G, Vejre H (2020). How can urban blue-green space be planned for climate adaption in high-latitude cities? A seasonal perspective. Sust. Cities Soc..

[39] Fan HY (2019). How to cool hot-humid (Asian) cities with urban trees? An optimal landscape size perspective. Agric. For. Meteorol..

[40] Tian P (2023). Assessing the cold island effect of urban parks in metropolitan cores: A case study of Hangzhou, China. Environ. Sci. Pollut. Res..

[41] Park CY (2019). Influence of urban form on the cooling effect of a small urban river. Landsc. Urban Plan..

[42] Yu ZW, Xu SB, Zhang YH, Jorgensen G, Vejre H (2018). Strong contributions of local background climate to the cooling effect of urban green vegetation. Sci. Rep..

[43] Cheng XY, Wei BS, Chen GJ, Li JX, Song CH (2015). Influence of park size and its surrounding urban landscape patterns on the park cooling effect. J. Urban Plan. Dev..

[44] Liao W, Cai ZW, Feng Y, Gan DX, Li XM (2021). A simple and easy method to quantify the cool island intensity of urban greenspace. Urban For. Urban Green..

[45] Peng J (2020). How to effectively mitigate urban heat island effect? A perspective of waterbody patch size threshold. Landsc. Urban Plan..

[46] Zhou S, Chen F, Xu Z (2022). Evaluating the accessibility of urban parks and waterfronts through online map services: A case study of Shaoxing, China. Urban For. Urban Green..

[47] Yang Z (2024). An assessment of urban park accessibility using multi-source data in Hefei, China: A social equity perspective. Land Degrad. Dev..

[48] Zha FK (2024). Understanding fine-scale heat health risks and the role of green infrastructure based on remote sensing and socioeconomic data in the megacity of Beijing, China. Ecol. Indic..

[49] Lu ZQJ (2010). The elements of statistical learning: Data mining, inference, and prediction, 2nd edition. J. R. Stat. Soc. Ser. A-Stat. Soc..

[50] Sun RH, Lü YH, Yang XJ, Chen LD (2019). Understanding the variability of urban heat islands from local background climate and urbanization. J. Clean Prod..

[51] Weng, Q. H., Liu, H., Liang, B. Q. & Lu, D. S. The spatial variations of urban land surface temperatures: Pertinent factors, zoning effect, and seasonal variability. *IEEE J. Sel. Top. Appl. Earth Observ. Remote Sens.* **1**, 154–166 10.1109/jstars.2008.917869 (2008).

[52] Zheng SX, Liu LC, Dong XF, Hu YQ, Niu PP (2022). Dominance of influencing factors on cooling effect of urban parks in different climatic regions. Int. J. Environ. Res. Public Health.

[53] Du HY (2019). Urban blue-green space planning based on thermal environment simulation: A case study of Shanghai, China. Ecol. Indic..

[54] Peng J, Jia JL, Liu YX, Li HL, Wu JS (2018). Seasonal contrast of the dominant factors for spatial distribution of land surface temperature in urban areas. Remote Sens. Environ..

