# Supplementary material for: A study of size threshold for cooling effect in urban parks and their cooling accessibility and equity
Source: Sci Rep. 2024 Jul 13;14:16176. doi: 10.1038/s41598-024-67277-2 (PMC11246519; doi:10.1038/s41598-024-67277-2)
Supplement: Supplementary file 1 — Supplementary Information. [file 41598_2024_67277_MOESM1_ESM.docx]

**A study of size threshold for cooling effect in urban parks and their cooling accessibility and equity: taking Harbin as an example**

**Jun Zhang, Huina Zhang * and Ruoming Qi**

College of Landscape Architecture, Northeast Forestry University, Harbin 150040, China

***** Correspondence: zhanghuina@nefu.edu.cn

**Supplementary materials**

**Table S1.** Analysis of variance (ANOVA) results.

| **Variable names** | **Cluster grouping** | **Sample size** | **Average value** | **Standard deviation** | **Variance test** | **Welch's test of variance** |
| --- | --- | --- | --- | --- | --- | --- |
| PCD | Bundle 1 | 19 | 0.397 | 0.132 | F=15.448 P=0.000*** | F=9.395 P=0.028** |
|  | Bundle 2 | 5 | 0.749 | 0.166 |  |  |
|  | Bundle 3 | 5 | 0.182 | 0.174 |  |  |
|  | Bundle4 | 2 | 0.68 | 0.127 |  |  |
| PCI | Bundle1 | 19 | 0.604 | 0.211 | F=7.541 P=0.001*** | F=11.057 P=0.002*** |
|  | Bundle2 | 5 | 0.61 | 0.075 |  |  |
|  | Bundle3 | 5 | 0.176 | 0.148 |  |  |
|  | Bundle4 | 2 | 0.578 | 0.014 |  |  |
| PCA | Bundle1 | 19 | 0.108 | 0.074 | F=38.527P=0.000*** | F=8.4 P=0.023** |
|  | Bundle2 | 5 | 0.74 | 0.25 |  |  |
|  | Bundle3 | 5 | 0.069 | 0.113 |  |  |
|  | Bundle4 | 2 | 0.116 | 0.04 |  |  |
| PCE | Bundle1 | 19 | 0.223 | 0.137 | F=28.516 P=0.000*** | F=71.193 P=0.000*** |
|  | Bundle2 | 5 | 0.081 | 0.083 |  |  |
|  | Bundle3 | 5 | 0.076 | 0.094 |  |  |
|  | Bundle4 | 2 | 0.954 | 0.065 |  |  |

The above table demonstrates the results of ANOVA including the results of mean ± standard deviation, F-test results and significance P-value. The P-values of the analysis of variance were all ≤ 0.05, indicating significant statistical differences among different bundles in PCD, PCI, PCA, and PCE.

**Table S2.** Reference table on the relationship between the Gini coefficient and equity.

| **Gini coefficient** | **Level of equity** |
| --- | --- |
| 0-0.2 | Absolute equality |
| 0.2-0.3 | Comparative equality |
| 0.3-0.4 | Relative reasonableness |
| 0.4-0.6 | Large disparity |
| 0.6-1 | Wide disparity |

**Table S3.** Ridge regression analysis results.

| **Cooling indices** | **Factors** | **Relative**  **contribution**  **(%)** | **Regression model** | **R^2^** | **F**  **(p)** |
| --- | --- | --- | --- | --- | --- |
| lnPCI | lnGrey | 100 | lnPCI=-0.47+0.143lnGrey | 0.503 | 2.235  (0.045) |
| lnPCD | lnNDVIin | 13 | lnPCD=2.43+0.113lnNDVIin+0.035lnBlue+0.086lnGreenout-  0.18lnGrey+0.082lnTR | 0.764 | 4.24  (0.003) |
|  | lnBlue | 16 |  |  |  |
|  | lnGreenout | 24 |  |  |  |
|  | lnGrey | 31 |  |  |  |
|  | lnTR | 16 |  |  |  |
| lnPCA | lnArea | 8 | lnPCA=1.022+0.082lnArea+0.181lnPerimete+0.509lnLSI+  0.0671lnGreenin+0.06lnBlue+0.142lnGreenout-0.244lnGrey+  0.131lnTR+0.045lnSH | 0.922 | 15.378  (0.000) |
|  | lnPerimeter | 11 |  |  |  |
|  | lnLSI | 8 |  |  |  |
|  | lnGreenin | 6 |  |  |  |
|  | lnBlue | 12 |  |  |  |
|  | lnGreenout | 17 |  |  |  |
|  | lnGrey | 19 |  |  |  |
|  | lnTR | 11 |  |  |  |
|  | lnSH | 8 |  |  |  |
| lnPCE | lnArea | 28 | lnPCE=1.87-0.26lnArea-0.291lnPerimete-0.117lnGreenin-  0.078lnGreenout+0.254lnGrey-0.141lnTR | 0.853 | 7.603  (0.000) |
|  | lnPerimeter | 18 |  |  |  |
|  | lnGreenin | 11 |  |  |  |
|  | lnGreenout | 10 |  |  |  |
|  | lnGrey | 20 |  |  |  |
|  | lnTR | 13 |  |  |  |

**Table S4.** Gini coefficient for cooling range accessibility for 103 neighborhoods.

| **No.** | **Neighborhood Name** | **Gini-public transportation** | **Gini-walk** | **Gini-average** |
| --- | --- | --- | --- | --- |
| 1 | Aijian | 0.1 | 0.1 | 0.1 |
| 2 | Anbu | 0.1 | 0.1 | 0.1 |
| 3 | Anhe | 0.1 | 0.1 | 0.1 |
| 4 | Anjing | 0.1 | 0 | 0.1 |
| 5 | Anle | 0.4 | 0.3 | 0.4 |
| 6 | Baoguo | 0.3 | 0.3 | 0.3 |
| 7 | Baojian Street | 0.4 | 0.3 | 0.3 |
| 8 | Binjiang | 0.2 | 0.2 | 0.2 |
| 9 | Chaoyang | 0.2 | 0.1 | 0.2 |
| 10 | Chenggaozi | 0 | 0.1 | 0.1 |
| 11 | Chengxiang Street | 0.1 | 0.2 | 0.2 |
| 12 | Chongjian | 0.1 | 0.1 | 0.1 |
| 13 | Dacheng | 0.4 | 0.3 | 0.3 |
| 14 | Daqing Street | 0.4 | 0.4 | 0.4 |
| 15 | Daxing | 0.1 | 0.1 | 0.1 |
| 16 | Dayoufang | 0.5 | 0.4 | 0.4 |
| 17 | Donglai | 0.1 | 0 | 0 |
| 18 | Dongyuan | 0.5 | 0.2 | 0.4 |
| 19 | Fendou Street | 0.5 | 0.4 | 0.4 |
| 20 | Fushui | 0.1 | 0.1 | 0.1 |
| 21 | Gexin | 0.2 | 0.2 | 0.2 |
| 22 | Gongcheng | 0.1 | 0.1 | 0.1 |
| 23 | Gongnong | 0.4 | 0.5 | 0.4 |
| 24 | Gongle | 0.2 | 0.2 | 0.2 |
| 25 | Haping Street | 0.6 | 0.5 | 0.5 |
| 26 | Haxi | 0.2 | 0.2 | 0.2 |
| 27 | Heping Street | 0.1 | 0.1 | 0.1 |
| 28 | Hexing Street | 0.4 | 0.4 | 0.4 |
| 29 | Hongqi | 0.3 | 0.2 | 0.3 |
| 30 | Hulan | 0.3 | 0.2 | 0.2 |
| 31 | Huayuan | 0.1 | 0.1 | 0.1 |
| 32 | Huagong | 0.1 | 0.1 | 0.1 |
| 33 | Huochetou | 0.2 | 0.1 | 0.1 |
| 34 | Jianguo | 0.4 | 0.4 | 0.4 |
| 35 | Jianshe Street | 0.1 | 0.2 | 0.1 |
| 36 | Jianzhu | 0.6 | 0.6 | 0.6 |
| 37 | Jiankang Street | 0.6 | 0.6 | 0.6 |
| 38 | Jinxiang | 0.6 | 0.6 | 0.6 |
| 39 | Jingwei | 0.2 | 0.2 | 0.2 |
| 40 | Jingyu | 0 | 0.1 | 0 |
| 41 | Kangan | 0.1 | 0.1 | 0.1 |
| 42 | Lanhe | 0.6 | 0.5 | 0.5 |
| 43 | Lihua | 0.2 | 0.2 | 0.2 |
| 44 | Liming | 0.3 | 0.3 | 0.3 |
| 45 | Limin | 0 | 0.1 | 0.1 |
| 46 | Lianmeng | 0.7 | 0.6 | 0.7 |
| 47 | Liaoyuan | 0.6 | 0.4 | 0.5 |
| 48 | Liushun | 0.4 | 0.3 | 0.4 |
| 49 | Lujia | 0.3 | 0.2 | 0.3 |
| 50 | Minqiang | 0.1 | 0.1 | 0.1 |
| 51 | Minsheng Street | 0 | 0.1 | 0.1 |
| 52 | Nanma | 0.1 | 0.1 | 0.1 |
| 53 | Nanshi | 0.1 | 0.1 | 0.1 |
| 54 | Nanzhi Street | 0.5 | 0.4 | 0.5 |
| 55 | Pingfang | 0.6 | 0.2 | 0.4 |
| 56 | Pingxin | 0.4 | 0.4 | 0.4 |
| 57 | Qizheng | 0.3 | 0.3 | 0.3 |
| 58 | Quxian | 0.1 | 0.1 | 0.1 |
| 59 | Qunli | 0.3 | 0.3 | 0.3 |
| 60 | Renli | 0.3 | 0.4 | 0.3 |
| 61 | Rongshi | 0.5 | 0.4 | 0.5 |
| 62 | Sankeshu | 0.1 | 0.1 | 0.1 |
| 63 | Shangzhi | 0.3 | 0.3 | 0.3 |
| 64 | Shengli | 0.3 | 0.2 | 0.2 |
| 65 | Shuini Street | 0 | 0 | 0 |
| 66 | Sidalin | 0.5 | 0.2 | 0.4 |
| 67 | Songbei | 0.4 | 0.4 | 0.4 |
| 68 | Songhuajiang | 0.1 | 0.1 | 0.1 |
| 69 | Songpu | 0.2 | 0.2 | 0.2 |
| 70 | Taigu | 0.1 | 0.1 | 0.1 |
| 71 | Taiping | 0.1 | 0.1 | 0.1 |
| 72 | Taiyangdao | 0 | 0 | 0 |
| 73 | Tiedong | 0.1 | 0 | 0.1 |
| 74 | Tongda | 0.1 | 0.1 | 0.1 |
| 75 | Tongjiang | 0.2 | 0.2 | 0.2 |
| 76 | Tongtian | 0.1 | 0.1 | 0.1 |
| 77 | Tongxiang | 0.1 | 0.1 | 0.1 |
| 78 | Tuanjie | 0.3 | 0.3 | 0.3 |
| 79 | Wanggang | 0.1 | 0.1 | 0.1 |
| 80 | Wangzhao | 0.2 | 0.1 | 0.1 |
| 81 | Wenhua | 0.1 | 0.1 | 0.1 |
| 82 | Wenzheng | 0.1 | 0.1 | 0.1 |
| 83 | Xianfeng Street | 0.4 | 0.3 | 0.3 |
| 84 | Xiangfang Street | 0.5 | 0.3 | 0.4 |
| 85 | Xiangfang Experimental Farm | 0.3 | 0.2 | 0.3 |
| 86 | Xincheng | 0.2 | 0.2 | 0.2 |
| 87 | Xinchun | 0 | 0.1 | 0.1 |
| 88 | Xinfa | 0.2 | 0.2 | 0.2 |
| 89 | Xinhua | 0.2 | 0.2 | 0.2 |
| 90 | Xinjiang | 0.3 | 0.3 | 0.3 |
| 91 | Xinle | 0.1 | 0.1 | 0.1 |
| 92 | Xinwei | 0.1 | 0.1 | 0.1 |
| 93 | Xinyang Street | 0.1 | 0 | 0.1 |
| 94 | Xinyi | 0 | 0.1 | 0 |
| 95 | Xingjian | 0.3 | 0.3 | 0.3 |
| 96 | Xingfu | 0.2 | 0.1 | 0.2 |
| 97 | Xueyuan Street | 0.3 | 0.4 | 0.4 |
| 98 | Yanjiagang Farm | 0.6 | 0.3 | 0.4 |
| 99 | Youxie | 0.8 | 0.2 | 0.5 |
| 100 | Yuejin | 0.3 | 0.2 | 0.2 |
| 101 | Zhaolin | 0.6 | 0.3 | 0.5 |
| 102 | Zhenjiang | 0.5 | 0.5 | 0.5 |
| 103 | Zhengyanghe | 0.2 | 0.2 | 0.2 |


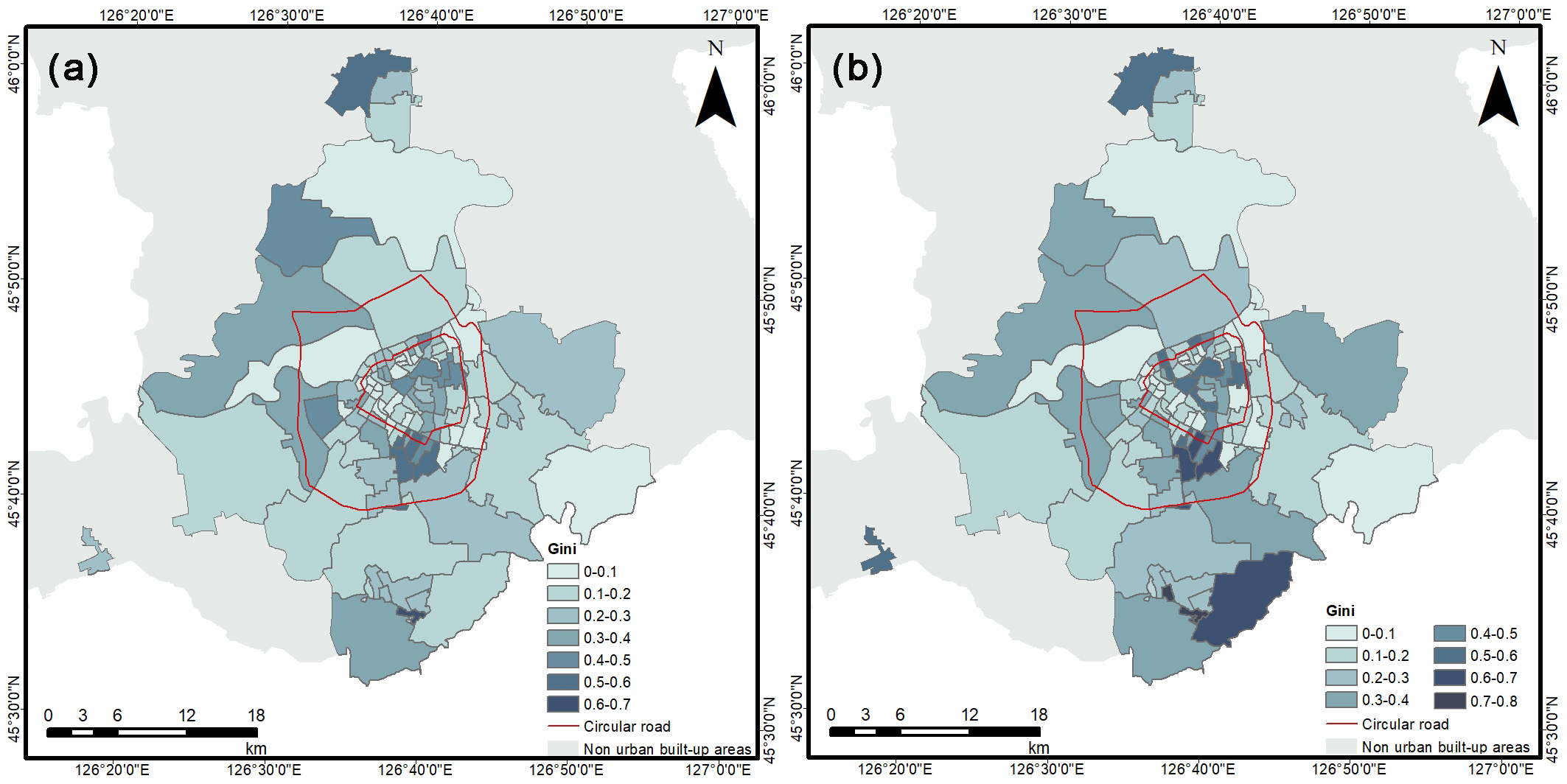


**Figure S1.** Spatial distribution of equality differences in park cooling services enjoyed by residents in 103 neighborhoods. (**a**) Equality of walking mode; (**b**) Equality of public transportation.

Note: Created using ArcGIS software. Neighborhood administrative boundaries were from BIGEMAP GIS Office software (http://www.bigemap.com/).
